# Prevalence and related factors of epilepsy in children and adolescents with cerebral palsy: a systematic review and meta-analysis

**DOI:** 10.3389/fped.2023.1189648

**Published:** 2023-07-28

**Authors:** Chao Gong, Annan Liu, Beibei Lian, Xixi Wu, Pei Zeng, Chaoli Hao, Bobo Wang, Zhimei Jiang, Wei Pang, Jin Guo, Shaobo Zhou

**Affiliations:** ^1^ College of Rehabilitation Medicine, Jiamusi University, Jiamusi, China; ^2^ Jiamusi University Affiliated No.3 Hospital, Jiamusi, China; ^3^ School of Science, Faculty of Engineering and Science, University of Greenwich, Medway Campus Central Avenue, Chatham Maritime, Kent, England

**Keywords:** cerebral palsy, epilepsy, prevalence, children, meta-analyses

## Abstract

**Objective:**

To study the worldwide prevalence and associated factors of epilepsy in children and adolescents with Cerebral Palsy (CP) and to analyze the differences between various subgroups.

**Method:**

We identified all potential studies on the prevalence of epilepsy in children and adolescents with CP from PubMed, Web of Science, and Embase. The search time was from the establishment of the database to November 2022. Randomized effects meta-analysis models were used to calculate the prevalence of epilepsy in CP. Subgroup analysis and meta-regression were utilized to further explore heterogeneity between articles and prevalence disparities between subgroups. The funnel plot and Egger's test were used to investigate potential publication bias.

**Results:**

Seventy-two articles, comprising 53,969 children and adolescents with CP, were included in this study. The results indicated a total epilepsy prevalence of 38.0% (95% CI: 34.8%–41.2%) in CP. The prevalence of epilepsy was 46.4% (95% CI: 41.4%–51.5%) in clinical sample-based studies and 31.6% (95% CI: 28.7%–34.5%) in population-based studies. Meta-regression demonstrated that the sample source, neonatal seizure, family history of epilepsy, EEG or cranial imaging abnormalities, intellectual/cognitive impairment, and topographical types of CP were heterogeneous contributors to the epilepsy prevalence in CP.

**Conclusion:**

Approximately one-third of children and adolescents with CP have epilepsy, and the sample source can significantly impact the total prevalence of epilepsy. Neonatal seizures, family history of epilepsy, EEG abnormalities, cranial imaging abnormalities, severe intellectual disability, and quadriplegia may be contributing factors to epilepsy comorbid in CP. Further study is required to verify the strength of these associations with epilepsy. This study aids in identifying the clinical characteristics of young people with CP at risk of developing epilepsy, which may assist clinicians in the early prevention and diagnosis of epilepsy within this population.

**Systematic Review Registration:** https://www.crd.york.ac.uk/PROSPERO/display_record.php?RecordID=367766, identifier CRD42022367766.

## 1. Introduction

Cerebral palsy (CP) is a group of permanent disorders of the development of movement and posture, causing activity limitation, that are attributed to non-progressive disturbances that occurred in the developing fetal or infant brain (1). The motor disorders of CP are often accompanied by disturbances of sensation, perception, cognition, communication, behaviour, epilepsy, and secondary musculoskeletal problems (1). According to population-based studies, the global prevalence of CP ranges between 0.1% and 0.4% (2), making it a common disorder threatening the early survival of children.

Epilepsy is an independent, significant, and common clinical problem in children with CP and is often regarded as an indicator of CP severity. The prevalence of epilepsy in the general population ranges from 0.4% to 0.8% (3). However it is significantly more prevalent in children with CP. Literature on epilepsy incidence in children with CP varies from 15% to 90%, with most estimates falling between 35% and 41%, making it five times higher than that of able-bodied children (4). This discrepancy may be attributed to CP and epilepsy sharing physiopathologic mechanisms caused by common etiology and risk factors. Many prenatal, peripartum, and postpartum injury, such as hypoxic-ischemic encephalopathy, infection, and congenital brain malformations, can cause CP and epilepsy (5). Epilepsy can be severe in some young people with CP, or it may be self-limiting and similar to other childhood epilepsies (6, 7). Nevertheless, epilepsy poses a significant threat to the early survival of young people with CP due to a variety of injuries and dysfunctions. The onset of epilepsy often indicates a more severe brain injury, affecting all functional aspects (8). Studies have shown that CP with epilepsy are often associated with severe intellectual disabilities, movement disorders, psychological and behavioral problems, and a lower quality of life, with less autonomy (9).

Many studies have shown that CP and epilepsy share a common etiologies and risk factors, and their co-occurrence rate is high. However, no systematic review and meta-analysis have yet been conducted on the prevalence of epilepsy in CP. Therefore, this systematic review aims to retrieve all published articles on the prevalence of epilepsy in children and adolescents with CP, estimate the epilepsy prevalence in CP using a meta-analysis, and analyze factors leading to epilepsy in CP, to provide evidence-based medical support.

## 2. Method

The protocol was registered with the International Prospective Register of Systematic Reviews (PROSPERO) (registration number: CRD42022367766).

### 2.1. Search strategy

Three databases (PubMed, Embase, and Web of Science) were systematically searched to determine the prevalence of epilepsy in children and adolescents with CP for all potentially relevant studies from database establishment to November 2022. The following search terms were used: cerebral pals*, CP, Little Disease, Spastic Diplegia, epileps*, seizure*, Aura, Convulsion, prevalence, epidemic, epidemiology, rate, morbidity, searched by combining MeSH Terms and Title/Abstract, combined according to Boolean logic principles (using AND, OR, or NOT). We used Endnote 20 to manage and remove repetitive articles. The reference lists of selected articles were also manually searched. In addition, articles on either CP or the prevalence of comorbidities with CP were searched in this systematic review. The search terms were presented in Supplementary S1.

### 2.2. Inclusion and exclusion criteria

#### 2.2.1. Inclusion criteria

(1) Articles that directly or indirectly reported the prevalence of CP with epilepsy. (2) English articles. (3) Articles containing original data: a cross-sectional study, a case-control study, and a cohort study. (4) Children and adolescents under the age of 19 with CP were included, in line with World Health Organization (WHO) criteria for the age of children and adolescents (10).

#### 2.2.2. Exclusion criteria

(1) Reviews, systematic reviews, meta-analyses, meeting summaries, student papers, comments, or letters. (2) To ensure that estimates reflect the expected population, articles with fewer than 50 participants were excluded. (3) The full text could not be obtained. (4) Article data were missing, repeated, or unavailable. (5) Articles on studying a certain type of CP population.

### 2.3. Article selection and data extraction

Two reviewers (CG and BBL) screened the retrieved articles by reading titles and abstracts according to the above criteria to exclude unrelated and repetitive articles, then read the full texts of the remaining publications independently. Finally, they discussed differences with a third expert (JG) to reach a consensus. The following data were extracted separately from each article: first author, year of publication, time of case collection, type of study, the total number of individuals with CP, number with epilepsy, prevalence, age, diagnostic criteria for epilepsy developed by the International League Against Epilepsy (ILAE), and proportion of females. If necessary, the study authors were contacted for more data or clarification. After discussion, the disagreement was resolved by consensus with the third senior reviewer (ZMJ).

### 2.4. Quality assessment

We used the Hoy et al. (11) tool to assess the risk of bias in studies measuring the prevalence of epilepsy in CP. The tool included ten items to judge the risk of bias: (1) selection-related bias, (2) bias associated with non-response, (3) measurement-related bias, and (4) bias associated with analysis. Each item included two options: (1) high or low risk. The high-risk option was chosen if the study lacked basic information to make the judgment. The overall assessment of the risk of bias was rated as low (three or fewer high-risk items), moderate (four to five high-risk items), and high (six or more high-risk items) risk bias. Two reviewers (XXW and PZ) individually reviewed each study. The disagreement was resolved by consensus and in consultation with the senior reviewer (WP). The details of Hoy were presented in Supplementary S2.

### 2.5. Data synthesis and analysis

Stata 17.0 (Stata Corp, College Station, TX, USA) was used to analyze the prevalence of epilepsy in children and adolescents with CP and provided its 95% confidence interval (CI). The Q test analyzed the heterogeneity among the included studies, and *I*^2^ quantitatively judged the heterogeneity. If the heterogeneity was high (*I*^2  ^≥  50%), the source of heterogeneity was further analyzed. After excluding the influence of articles with obvious heterogeneity, the random effect model (REM) was used for Meta-analysis. Otherwise, the fixed effect model (FEM) was used. Subgroup analysis and Meta-regression analysis were used to explore the sources of heterogeneity and the differences in the prevalence of epilepsy among subgroups.

According to the sample source in the articles, we divided the articles into two groups: a clinical sample-based study group and a population-based study group. Clinical sample-based studies considered individuals with CP from hospitals, clinics, or rehabilitation institutions. Population-based studies were considered individuals with CP in the entire population or a random sampling population. Subgroup analysis was performed on the gender, ethnic group, study type, national income level, diagnostic criteria for epilepsy developed by the International League Against Epilepsy (ILAE), and publication time of the two groups to explore the sources of heterogeneity and the differences in the prevalence of epilepsy between different groups.

In addition, we performed subgroup analysis and meta-regression analysis based on the characteristics of children with CP to explore the differences in the prevalence of epilepsy between different groups. The related factors included gestational age, birth weight, birth asphyxia, neonatal seizures (NS), family history of epilepsy, central nervous system (CNS) abnormalities based on MRICS (Magnetic Resonance Imaging Classification System), electroencephalography (EEG) abnormalities, cranial imaging abnormalities, cognitive/intellectual status, clinical type of CP and topographical type of CP.

## 3. Results

### 3.1. Article selection

**Figure 1:**
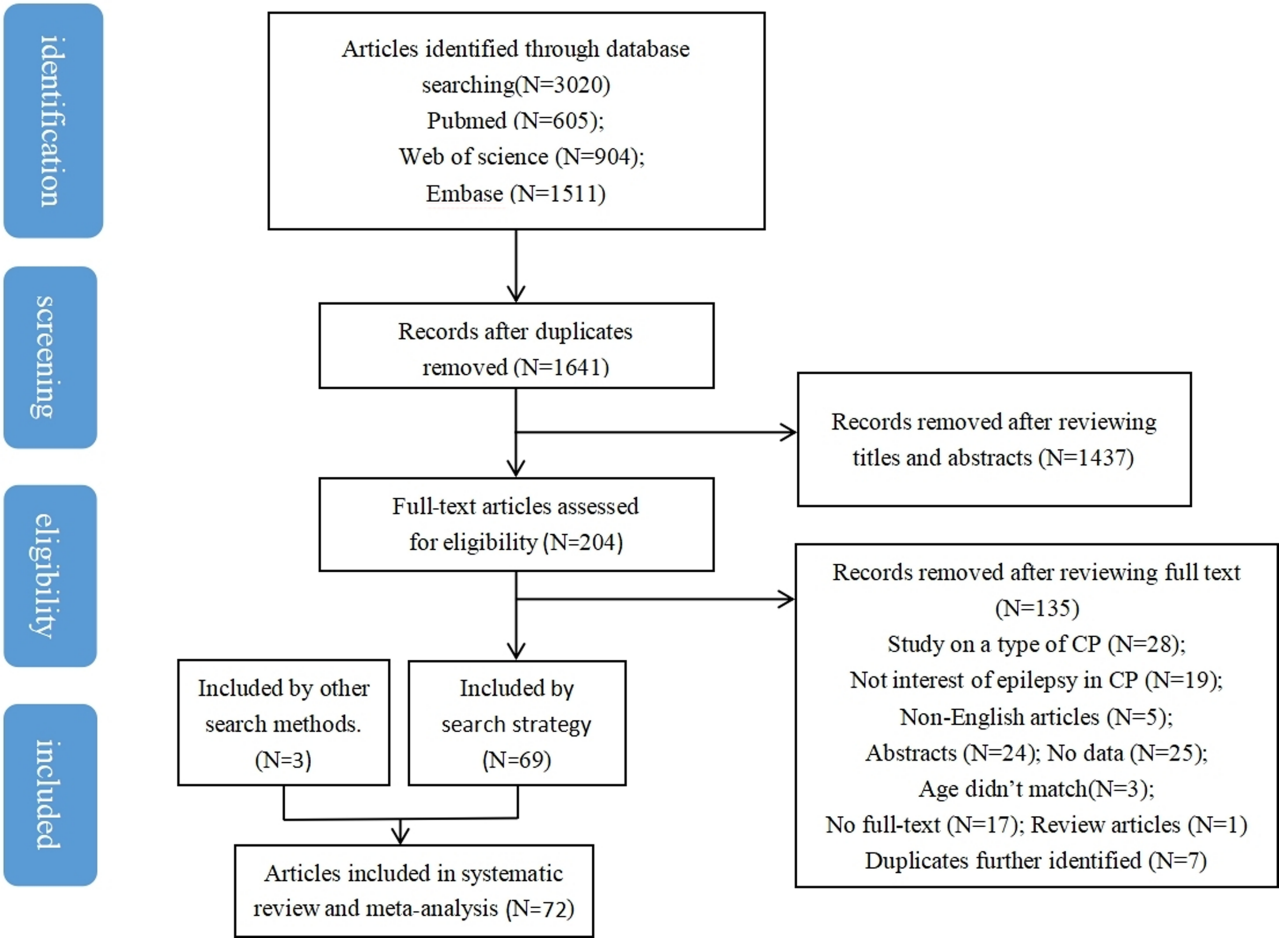
Article screening process and result.

3,020 articles were retrieved: PubMed: 605, Embase: 904, Web of Science: 1,511. After removing duplicates, 1,641 articles remained. After title and abstract screening, 204 full-texts were evaluated for eligibility according to inclusion and exclusion criteria, 69 articles were included. In addition, 3 articles were included by other search methods. Therefore, a total of 72 articles were included in this study. The article selection process is shown in Figure 1.

### 3.2. Article characteristic

The 72 articles included in this meta-analysis were published from 1998 to 2022, involving 38 countries and regions worldwide, and the data were collected from 1988 to 2019. There were 32 articles based on clinical samples and 40 articles based on population. According to the main ethnic composition of the country, the ethnic groups included are Europa (43 articles), Mongolian (8 articles), Black (11 articles), and multi-ethnic mixed (10 articles). The number of children with CP included in the article ranged from 62 to 9,654, totaling 53,969 people. The quality scores of the articles ranged from 1 to 5 points, of which 52 were low and 20 were moderate. A detailed description of the articles is shown in Table 1. The detailed quality assessment results are shown in Supplementary S3.

**Table 1:** Summary of articles.

First author	Published time	Countries or regions	Main ethnic composition	Time range of data collection	CP	Epilepsy	Prevalence (%)	Age (years), M (SD)	Proportion of female (per 100)	Diagnostic criteria of epilepsy	Quality score
Come from clinical samples-based study											
Kwong (12)	1998	China	Mongolian ethnic group	1990–1995	85	32	37.6	2–17.5	43.5	ILAE in 1981,1989 and 1993	3
Zafeiriou (13)	1999	Greece	European ethnic group	–	493	178	36.1	–	–	ILAE in 1981,1989 and 1993	3
Bruck (14)	2001	Brazil	Multi-ethnic group	1996–1998	100	62	62	2–18	49	ILAE in 1989 and 1993	3
Sianturi (15)	2002	Indonesia	Mongolian ethnic group	1988.07–1998.06	67	25	37.3	0.5–14.5	52.2	ILAE in 1981	3
Şenbil (16)	2002	Turkey	European ethnic group	1997–1999	74	31	41.8	2–10	41.8	ILAE in 1989 and 1993	3
Singhi (17)	2003	India	European ethnic group	–	452	160	35.3	1–14	–	ILAE in 1981,1989 and 1993	3
Kulak (18)	2003	Poland	European ethnic group	1994.10–2001.03	198	82	41.4	1–18	35.4	ILAE in 1989	4
Zelnik (19)	2011	Israel	European ethnic group	2005–2006	197	65	32.9	7.2 (3.0); 1.4–12.8	40.6	ILAE in 1989	4
Mert (20)	2011	Turkey	European ethnic group	2006.11–2009.10	98	56	57.1	3–16	42.8	ILAE in 1989	3
Frank-Briggs (21)	2011	Nigeria	Black ethnic group	2008.06–2010.06	834	418	50.1	<10	35.73	–	3
Prasad (8)	2011	India	European ethnic group	2007.09–2009.03	102	53	51.9	<15	23.5	–	4
Aronu (22)	2013	Nigeria	Black ethnic group	2007.01.01–2009.12.31	164	62	37.8	–	43.9	ILAE in 1981	3
Kakooza-Mwesige (23)	2015	Uganda	Black ethnic group	2009.09–2010.08	135	61	45.1	2–12	46.6	–	3
Keeratisiroj (24)	2015	Thailand	Mongolian ethnic group	2008–2013	527	246	46.7	2–18	–	–	3
Bearden (25)	2016	Botswana	Black ethnic group	2013–2014	68	52	76.4	2–18	–	–	4
Minocha (26)	2017	India	European ethnic group	2012.3–2013.3	180	75	41.6	1–12	43.3	–	4
Gincota (27)	2018	Moldova	European ethnic group	2016.6–2016.9	185	93	50.2	6.6–7.6	36.2	ILAE in 1993	3
Gürkan (28)	2018	Turkey	European ethnic group	2008.01–2015.12	234	118	50.4	1–8.7	41	ILAE in 1993	4
Sucuoğlu (29)	2018	Turkey	European ethnic group	2015.07–2017.08	204	66	32.3	10.35 (8.32)	42.2	–	4
Aydin (30)	2019	Turkey	European ethnic group	–	197	102	51.7	1–18	41.6	–	4
Karatoprak (31)	2019	Turkey	European ethnic group	2002.12–2012.12	234	126	53.8	3–18	41.4	ILAE in 1981	3
Hanci (32)	2020	Turkey	European ethnic group	2016.11–2019.11	229	120	52.4	1–13	44.9	ILAE in 2017	3
Pavone (33)	2020	Italy	European ethnic group	2008.05–2018.11	93	46	49.4	1–10	52.5	–	4
Sadowska (34)	2020	Poland	European ethnic group	2008–2016	181	102	56.3	4–17	45.8	–	3
Al-Blowi (35)	2020	Saudi arabia	European ethnic group	2012–2016	119	95	79.8	1–12	52.9	–	5
Şik (36)	2021	Turkey	European ethnic group	2012.01–2015.09	62	26	41.9	9.31 ± 4.13, 3–18	32.3	–	2
Tsige (37)	2021	Ethiopia	Black ethnic group	2018.07–2018.09	174	106	60.9	2–18	–	–	4
Jibril (38)	2021	Nigeria	Black ethnic group	2018.09–2019.03	165	56	33.9	1–12	32.7	–	3
Chaudhary (39)	2022	Nepal	Multi-ethnic group	2017.03–2018.03	110	73	66.3	0.5–15	25.4	ILAE in 2014	4
Mangamba (40)	2022	Cameroon	Black ethnic group	2017.12.1–2018.5.31	198	68	34.3	0.25–15	45.9	ILAE in 2014	5
K, Archana (41)	2022	India	European ethnic group	2018.01–2019.07	300	79	26.3	1–12	31	ILAE in 2017	4
Karim (42)	2022	Vietnam	Mongolian ethnic group	2017.06–2017.11	765	82	10.8	<18	35.8	–	5
Comes from population-based study											
Aneja (43)	2001	India	European ethnic group	–	831	121	14.5	–	–	ILAE in 1981	4
Nordmark (44)	2001	Sweden	European ethnic group	NA-1998	167	61	36.5	5–8	45.4	–	3
Parkes (45)	2001	Northern Ireland	European ethnic group	NA-2000.2	710	155	21.8	7–19	–	–	3
Carlsson (46)	2003	Sweden	European ethnic group	2000.11–2001.05	146	55	37.6	6–14	43.8	ILAE in 1981,1989 and 1993	4
El-Tallawy (47)	2007	Egypt	European ethnic group	–	98	48	48.9	–	46.9	ILAE in 1981	3
Beckung (48)	2007	Sweden	European ethnic group	–	176	62	35.2	5–8	–	–	2
Andersen (49)	2008	Norway	European ethnic group	2003.01.01–2006.03.31	294	80	27.2	1.9–10.2	49.3	ILAE in 1993	3
Sigurdardóttir (50)	2009	Iceland	European ethnic group	–	139	37	26.6	–	53.2	–	2
Kirby (51)	2011	The United States	Multi-ethnic group	NA-2006	476	166	34.8	8	45.4	ILAE in 1993	3
Himmelmann (52)	2011	Sweden	European ethnic group	–	186	82	44	4–8	48.3	ILAE in 1993	2
Sellier (53)	2012	European countries	European ethnic group	–	9,654	3,424	35.4	–	41.8	ILAE in 1993	4
Singhi (54)	2013	India	European ethnic group	2000–2009	1,212	539	44.4	<8	38.9	–	3
Christensen (55)	2014	The United States	Multi-ethnic group	NA-2008	451	185	41	8	40	ILAE in 1993	3
Yalcinkaya (56)	2014	Turkey	European ethnic group	–	730	167	22.9	7.27 (4.63); 2–18	41.2	–	2
Granild-Jensen (57)	2015	Danish	European ethnic group	–	1,222	356	29.1	–	40.8	–	3
Meehan (58)	2015	Australia	Multi-ethnic group	2008–2012	2,099	623	29.6	<19	–	ILAE in 1993	2
Delacy (59)	2016	Australia	Multi-ethnic group	2013	3,173	883	27.8	8–18	–	ILAE in 1993	2
Tan (60)	2016	Netherlands	European ethnic group	2002–2007	309	74	23.9	1–15	37.6	–	2
Khandaker (61)	2016	Bangladesh	Multi-ethnic group	2015.01–2015.06	299	88	29.4	<18		–	1
Delobel-Ayoub (62)	2017	European countries	European ethnic group	–	1,225	374	30.5	0–19	–	–	1
Abas (63)	2017	Egypt	European ethnic group	2015.5–2015.11	200	76	38	0.25–18	45	–	1
Kakooza-Mwesige (64)	2017	Uganda	Black ethnic group	2015.03.01–2015.6.30	360	77	21.3	2–17	–	–	3
Yim (65)	2017	Korea	Mongolian ethnic group	NA-2015.01	773	243	30.3	–	42.8	–	3
Hollung (66)	2018	Norway	European ethnic group	1999–2010	1,664	383	23	–	–	ILAE in 1993	1
Reid (67)	2018	Australia	Multi-ethnic group	–	1,005	299	29.7	–	41.7	ILAE in 1993	4
Chiang (68)	2019	Taiwan, China	Mongolian ethnic group	2010–2011	8,419	2,510	29.8	<19	–	–	3
Jonsson (69)	2019	Sweden	European ethnic group	–	205	70	34.1	–	40.9	–	3
Khandaker (70)	2019	Bangladesh	Multi-ethnic group	2015.01–2016.12	726	170	23.4	<18	38.2	–	1
Påhlman (71)	2019	Sweden	European ethnic group	2017.1–2017.3	264	109	41.2	10–17	46.5	–	1
Andrews (72)	2019	Uganda	Black ethnic group	NA-2015	93	33	35.4	2–17	43.2	–	2
Rafique (73)	2020	Pakistan	European ethnic group	2010–2016	658	96	14.5	–	41.7	–	3
Jahan (74)	2020	Indonesia	Mongolian ethnic group	2017.3–2017.8	123	15	12.1	0–18	43.8	–	4
Hollung (75)	2020	Norway	European ethnic group	2008–2017	2,302	898	39	–	42.1	–	1
Yamagishi (76)	2021	Japan	Mongolian ethnic group	2017.4–2018.3	135	46	34	4–8	–	–	3
Duke (77)	2021	Nigeria	Black ethnic group	2017.12–2018.07	388	130	33.5	4–15	40.9	ILAE in 2014	1
Al-Garni (78)	2021	Saudi arabia	European ethnic group	2014–2019	679	196	28.8	0.7–17.8	43.1	–	1
Bambi (79)	2021	Uganda	Black ethnic group	2017.11–2018.05	224	138	61.6	2–12	37.5	–	3
Karim (80)	2022	Bangladesh	Multi-ethnic group	2015.01–2016.12	726	170	23.4	<18	–	ILAE in 2017	1
Szpindel (81)	2022	Canada	European ethnic group	1999.01.01–2012.12.31	302	127	42	10–13	44.3	–	1
Linton (82)	2022	Sweden	European ethnic group	NA-2018	3,902	1,291	33	3–18	42.3	–	1

### 3.3. Prevalence of epilepsy

**Figure 2:**
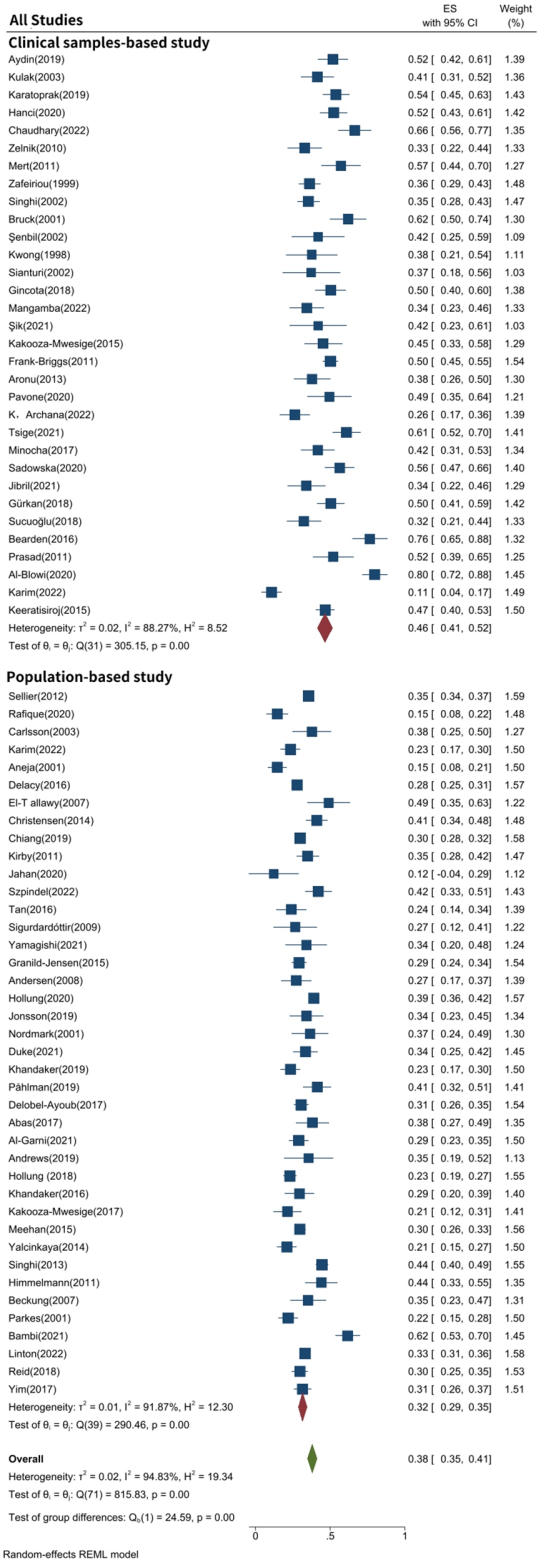
Forest plot of meta-analysis of prevalence of epilepsy in CP.

As shown in Figure 2, the total prevalence of epilepsy in CP was 38.0% (95% CI: 34.8%–41.2%), and significant heterogeneity was found in the study *I*^2  ^=  94.8%, *P *<  0.001). Table 2 shows a statistically significant difference between clinical sample-based and population-based studies (*P *<  0.001). As shown in Figure 2 and Table 2, 32 articles were clinical sample-based studies. The prevalence of epilepsy was 46.4% (95% CI: 41.4%–51.5%), with significant heterogeneity (*I*^2 ^= 88.3%, *P *< 0.001). As shown in Figure 2, 40 articles were population-based studies. The prevalence of epilepsy was 31.6% (95% CI: 28.7%–34.5%), with significant heterogeneity (*I*^2 ^= 91.9%, *P *< 0.001).

**Table 2:** According to the sample source in the articles, the estimated prevalence of epilepsy in CP.

Subgroup	Number of studies	*N*	Event	*I* ^2^	*P* value	(95% CI)		Prevalence (%)	Results of meta-regression for prevalence of CP		
						Low	High		*P*-value	Exp (*b*)	95% CI
Clinical sample-based study	32	7,224	3,016	88.3	<0.001	0.414	0.515	46.4	<0.001	0.8144525	0.8043876 to 0.912799
Population-based study	40	46,745	14,643	91.9	<0.001	0.287	0.345	31.6			

### 3.4. Subgroup analysis of clinical sample-based studies

As shown in Table 3, the prevalence of epilepsy in males with CP was 47.4%, and the prevalence of epilepsy in females with CP was 49.2%. The prevalence of epilepsy in 1981, 1989, and 1993 ILAE was 44.3%, and the majority of epilepsy in 2014 and 2017 ILAE was 44.9%. According to the ethnic group, the prevalence of epilepsy in the European ethnic group was 46.6%, the prevalence of epilepsy in the Mongolian ethnic group was 32.8%, the prevalence of epilepsy in the Black ethnic group was 48.6%, and the prevalence of epilepsy in the multi-ethnic group was 64.4%. According to the national income level, the prevalence of epilepsy in low-and middle-income countries was 47.0%, and the prevalence of epilepsy in high-income countries was 43.0%. The prevalence of epilepsy in CP was 39.9% from 1998 to 2003, 50.2% from 2011 to 2016, and 46.7% from 2017 to 2022.

**Table 3:** The estimated prevalence of epilepsy in CP based on clinical sample studies.

Subgroup	Number of studies	*N*	Event	*I* ^2^	*P* value	(95% CI)		Prevalence (%)	*P* value between groups
Gender									
Female	12	784	374	26.0	0.188	0.433	0.551	49.2	0.760
Male	12	1,109	504	56.8	0.008	0.407	0.542	47.4	
Diagnostic criteria for epilepsy developed by the ILAE									
1981, 1989 and 1993	13	2,581	1,090	67.2	<0.001	0.390	0.496	44.3	0.940
2014 and 2017	4	837	340	91.5	<0.001	0.275	0.622	44.9	
Ethnic group									
Europoid	19	3,832	1,673	85.8	<0.001	0.404	0.529	46.6	0.329
Mongoloid	4	1,444	385	95.0	<0.001	0.108	0.548	32.8	
Black	7	1,738	823	86.3	<0.001	0.387	0.586	48.6	
Multi-ethnic mixed	2	210	135	0	=0.598	0.564	0.725	64.4	
National income level									
Low-and Middle-income	27	6,062	2,543	90.9	<0.001	0.408	0.533	47.0	0.588
High-income	5	1,162	473	72.7	0.005	0.342	0.517	43.0	
Publication time									
1998–2003	7	1,469	570	61.7	0.016	0.359	0.438	39.9	0.587
2011–2016	7	1,598	767	80.0	<0.001	0.408	0.597	50.2	
2017–2022	18	4,157	1,679	92.9	<0.001	0.382	0.552	46.7	

### 3.5. Subgroup analysis of population-based studies

**Figure 3:**
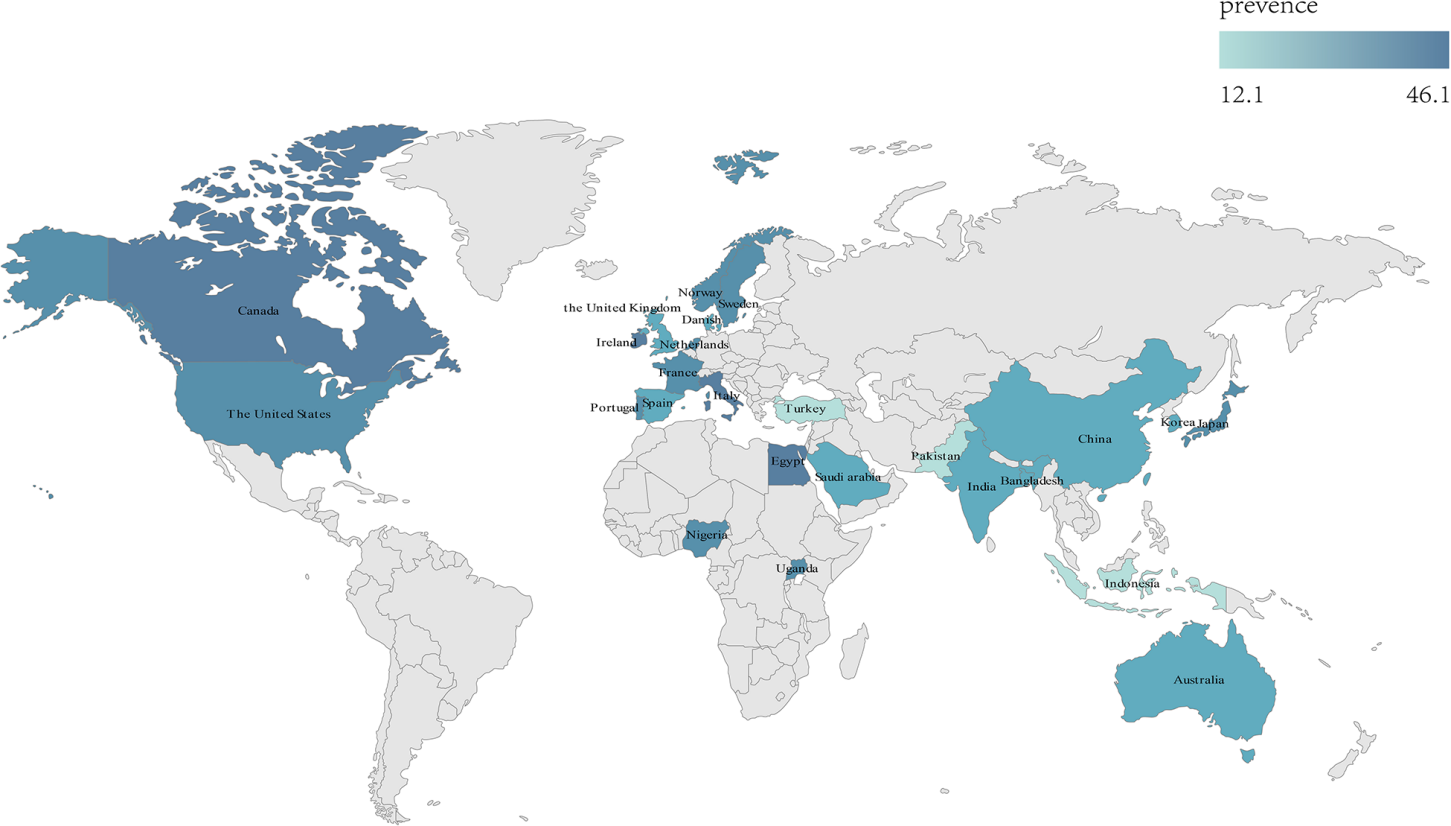
Prevalence of epilepsy in CP in different countries or regions in population-based study.

As shown in Table 4, the prevalence of epilepsy in males with CP was 36.2%, and the prevalence of epilepsy in females with CP was 31.4%. The prevalence of epilepsy in 1981, 1989, and 1993 ILAE was 33.2%, and the majority of epilepsy in 2014 and 2017 ILAE was 32.4%. According to the ethnic group, the prevalence of epilepsy in the European ethnic group was 31.6%, the prevalence of epilepsy in the Mongolian ethnic group was 29.6%, the prevalence of epilepsy in the Black ethnic group was 38.1%, and the prevalence of epilepsy in the multi-ethnic group was 29.6%. According to the national income level, the prevalence of epilepsy in low- and middle-income countries was 30.7%, and that in high-income countries was 32.1%. The prevalence of epilepsy in CP was 26.4% from 1998 to 2003, 34.0% from 2004 to 2010, 32.7% from 2011 to 2016, and 31.5% from 2017 to 2022. The distribution of epilepsy prevalence is shown in Figure 3.

**Table 4:** The estimated prevalence of epilepsy in CP based on population studies.

Subgroup	Number of studies	*N*	Event	*I* ^2^	*P* value	(95% CI)		Prevalence (%)	*P* value between groups
Gender									
Female	5	4,342	1,463	74.6	0.003	0.212	0.417	31.4	0.586
Male	5	5,998	1,972	83.4	<0.001	25.7	46.6	36.2	
Diagnostic criteria for epilepsy developed by the ILAE									
1981, 1989 and 1993	12	20,715	6,864	87.4	<0.001	29.3	37.0	33.2	0.447
2014 and 2017	2	1,114	300	72.8	0.55	28.8	36.0	32.4	
Ethnic group									
Europoid	24	27,275	8,867	87.6	<0.001	0.284	0.348	31.6	0.909
Mongoloid	4	9,450	2,814	40.2	0.171	0.253	0.338	29.6	
Black	4	1,065	378	93.5	<0.001	0.192	0.570	38.1	
Multi-ethnic mixed	8	8,955	2,584	65.5	0.005	0.263	0.329	29.6	
National income level									
Low- and middle-income	15	15,643	4,545	92.0	<0.001	0.251	0.363	30.7	0.619
High-income	25	31,102	10,098	77.2	<0.001	0.298	0.344	32.1	
Publication time									
1998–2003	4	1,854	392	82.0	<0.001	0.161	0.367	26.4	0.597
2004–2010	4	707	227	58.1	0.067	0.245	0.435	34.0	
2011–2016	11	19,811	6,573	87.6	<0.001	0.288	0.367	32.7	
2017–2022	21	24,373	7,451	86.9	<0.001	0.281	0.348	31.5	

### 3.6. Based on the characteristics of CP to explore the subgroup factors related to comorbid epilepsy in CP

As shown in Table 5, in terms of gestational age, the prevalence of epilepsy in preterm CP (36.4%, 95% CI: 28.4%–44.4%) was lower than in term birth (45.7%, 95% CI: 38.9%–45.7%). Regarding birth weight, the prevalence of epilepsy in low birth weight CP (44.9%, 95% CI: 30.4%–59.4%) was lower than that in normal weight children (53.4%, 95% CI: 38.7%–68.0%). Regarding birth asphyxia, the prevalence of epilepsy in CP with birth asphyxia (46.6%, 95% CI: 33.4%–59.8%) was higher than that without birth asphyxia (41.7%, 95% CI: 38.1%–52.0%). Regarding NE, the prevalence of epilepsy in CP with NE (66.9%, 95% CI: 56.6%–77.2%) was significantly higher than that without NE (42.1%, 95% CI: 35.3%–48.8%) (*P *< 0.001). Regarding a family history of epilepsy, the prevalence of epilepsy in CP with a family history of epilepsy (89.2%, 95% CI: 81.1%–97.3%) was significantly higher than that without a family history of epilepsy (49.0%, 95% CI: 44.4%–53.7%) (*P *< 0.001). Regarding EEG abnormalities, the prevalence of epilepsy in CP with EEG abnormalities (60.8%, 95% CI: 45.3%–76.3%) was significantly higher than that without EEG abnormalities (24.0%, 95% CI: 6%–42.0%) (*P* = 0.009). Regarding cranial imaging abnormalities, the prevalence of epilepsy in CP with cranial imaging abnormalities (60.8%, 95% CI: 57.8%–37.2%) was significantly higher than that without cranial imaging abnormalities (36.3%, 95% CI: 24.3%–48.3%) (*P* = 0.009).

**Table 5:** Subgroup analysis of CP comorbid epilepsy based on the characteristics of CP.

Subgroup	Number of studies	*N*	Event	*I* ^2^	*P* value	(95% CI)		Prevalence (%)	Results of meta-regression for prevalence of CP		
									*P*-value	Exp (*b*)	95% CI
Gestational age											
<37 w	13	4,862	1,405	78.7	<0.001	0.284	0.444	36.4	0.086	1.095615	0.9863146 to 1.217028
≥37 w	13	5,755	2,306	73.7	<0.001	0.389	0.457	45.7			
Birth weight											
<2,500 g	5	4,267	1,242	88.2	<0.001	0.304	0.594	44.9	0.331	1.092679	0.8939114 to 1.335644
≥2,500 g	4	4,928	1,969	90.7	<0.001	0.387	0.680	53.4			
Neonatal seizures											
Yes	12	742	437	78.8	<0.001	0.566	0.772	66.9	<0.001	0.7750931	0.6820871 to 0.8807809
No	12	5,485	1,885	80.6	<0.001	0.353	0.488	42.1			
Birth asphyxia											
Yes	3	307	146	58.5	0.09	0.334	0.598	46.6	0.446	1.063259	0.8689285 to 1.301051
No	3	284	118	0.0	0.866	0.381	0.520	41.7			
Family history of epilepsy											
Yes	6	63	52	0.0	<0.001	0.811	0.973	89.2	<0.001	0.6752944	0.5988776 to 0.7614619
No	6	910	439	0.0	<0.001	0.444	0.537	49.0			
EEG abnormalities											
Yes	3	487	274	84.4	0.002	0.453	0.763	60.8	0.009	0.684606	0.4567173 to 1.026204
No	3	90	21	0.0	0.731	0.06	0.420	24.0			
Cranial imaging abnormalities											
Yes	8	801	450	63.4	0.008	0.502	0.655	57.8	0.009	0.8061711	0.6925094 to 0.9384882
No	8	342	110	43.5	0.088	0.243	0.483	36.3			
CNS pathological changes based on MRICS											
Predominantly White matter injuries	3	104	56	4.7	0.350	0.428	0.69	55.9	0.127	0.9707238	0.9336145 to 1.009308
Predominantly White matter injuries	3	219	94	53.9	0.114	0.153	0.577	36.5			
Malformations	5	730	385	0.0	0.939	0.479	0.578	52.8			
Miscellaneous	3	94	39	25.0	0.264	0.256	0.620	43.8			
Normal	6	247	95	2.1	0.403	0.308	0.504	40.6			
Cognitive/intellectual status											
Normal	8	927	159	5.2	0.390	0.121	0.238	17.9	<0.001	1.206163	1.161264 to 1.252798
Mild	4	139	53	0.0	0.914	0.215	0.515	38.5			
Moderate	3	105	70	0.0	0.577	0.568	0.786	67.7			
Severe	4	237	175	5.5	0.365	0.688	0.815	75.2			
Clinical type of CP											
Spastic type	16	10,482	3,277	80.1	<0.001	0.374	0.480	42.7	0.528	1.013248	0.9720165 to 1.056228
Dyskinetic type	19	1,185	582	55.0	0.002	0.447	0.611	52.9			
Ataxia type	15	760	237	41.5	0.047	0.290	0.491	39.1			
Mixed type	9	284	129	60.0	0.010	0.364	0.643	45.9			
Topographical type of CP											
Quadriplegia	21	2,367	1,350	62.1	<0.001	0.570	0.665	61.8	<0.001	0.8863164	0.8414422 to 0.9335837
Diplegia	21	2,049	476	13.7	0.280	0.216	0.309	26.3			
Hemiplegia	21	1,879	570	72.0	<0.001	0.318	0.487	40.2			

Regarding CNS abnormalities based on MRICS, the prevalence from high to low was predominantly gray matter injuries (55.9%, 95% CI: 42.8%–69.0%), brain malformation (52.8%, 95% CI: 47.9%–57.8%), miscellaneous (43.8%, 95% CI: 47.9%–57.8%), normal (40.6%, 30.8%–50.4%) and predominantly white matter injuries (36.5%, 95% CI: 15.3%–57.7%). Regarding cognition/intelligence, the prevalence significantly increased with the degree of cognition/intelligence, followed by normal (17.9%, 95% CI: 12.1%–23.8%), mild (38.5%, 95% CI: 21.5%–51.5%), moderate (67.7%, 95% CI: 56.8%–78.6%), severe (75.2%, 95% CI: 68.8%–81.5%) (*P* < 0.001). Regarding the clinical type of CP, the prevalence from high to low was the dyskinetic type (52.9%, 95% CI: 44.7%–61.1%), mixed type (45.9%, 95% CI: 36.4%–64.3%), spastic type (42.7%, 95% CI: 37.4%–48.0%) and ataxia type (39.1%, 95% CI: 29.0%–49.1%). Regarding the topographical type of CP, the prevalence from high to low was quadriplegia (61.8%, 95% CI: 57.0%–66.5%), hemiplegia (40.2%, 95% CI: 31.8%–48.7%), and diplegia (26.3%, 95% CI: 21.6%–30.9%) (*P* < 0.001).

### 3.7. Publication bias

**Figure 4:**
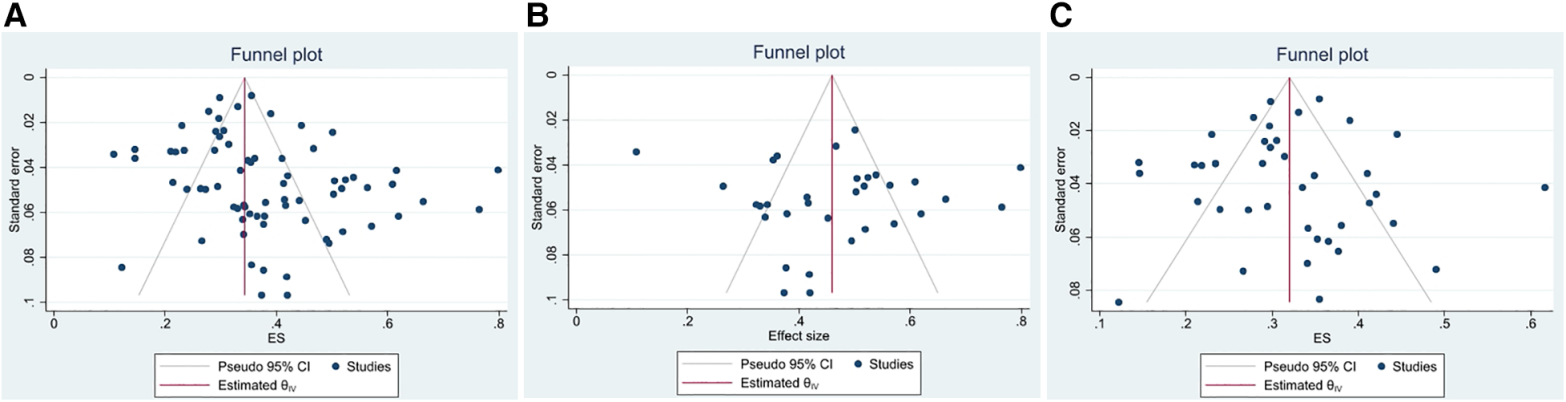
The funnel plot of the total prevalence of epilepsy in CP. (A) All studies (B) Clinical sample-based study (C) Population-based study.

**Figure 5:**
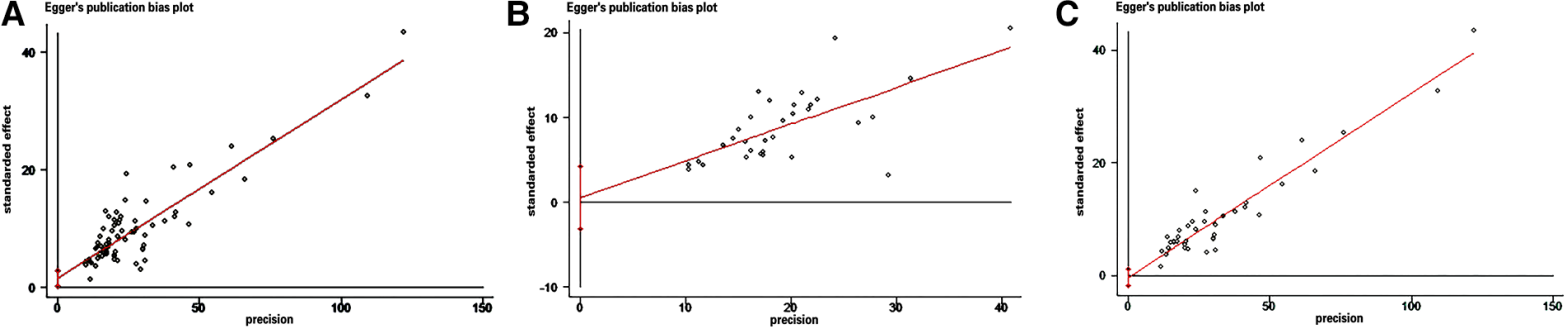
Egger's test of the prevalence of epilepsy in CP. (A) All studies (B) Clinical sample-based studies (C) Population-based studies.

The funnel plot was used to assess the presence of publication bias, revealing an evident asymmetry in the overall prevalence of epilepsy. However, we conducted separate analyses by sample source, resulting in symmetrical funnel plots for both clinical case-based and population-based studies (see Figure 4). The application of Egger's test indicated a significant publication bias in the meta-analysis (*t* = 2.39, *P* < 0.05). Further analysis utilizing Egger's test based on sample source revealed no significant publication bias in the clinical sample-based study (*t* = 0.34, *P* = 0.733) and population-based study (*t* = −0.50, *P* = 0.623), as depicted in Figure 5.

### 3.8. Sensitivity analysis and meta-regression analysis

A sensitivity analysis was conducted using the single-article removal method, and no studies were found to affect the overall results significantly. Meta-regression analysis was then performed on the included articles, with the prevalence of epilepsy in CP as the dependent variable. Independent variables for the meta-regression analysis included the sample source of the articles, gestational age, birth weight, birth asphyxia, NE, family history of epilepsy, CNS abnormalities based on MRICS, EEG abnormalities, cranial imaging abnormalities, cognitive/intellectual status, clinical type of CP, and topographical type of CP. The results revealed significant heterogeneity among the sample source of articles, NE, family history of epilepsy, EEG abnormalities, imaging abnormalities, intellectual/cognitive impairment, and topographical type of CP (*P* < 0.05), as illustrated in Tables 2, 5.

## 4. Discussion

The total prevalence of epilepsy in CP in this study was 38.0%, and epilepsy in children with CP was 46.4% in a clinical case-based study and 31.6% in a population-based study. The number of individuals with CP in included articles was enough to represent the prevalence of epilepsy in CP, which can reflect the prevalence of epilepsy in CP to some extent. In clinical sample-based studies, the prevalence of epilepsy in CP was generally high, which might be caused by the individuals were mainly from medical institutions, or the individuals admitted were generally more severe. They are more likely to have comorbidities such as intellectual disability, epilepsy, and speech disorders, leading to overestimating the prevalence of epilepsy in CP (83). In contrast, CP with milder symptoms might not go to hospitals or rehabilitation facilities for rehabilitation, thus missing some individuals with mild to moderate CP that were less likely to develop epilepsy. In population-based studies, the prevalence of CP at the national level was unknown because some countries or regions need a specific population-based national registry (48). Therefore the studies only covered part of the region and represented the prevalence of epilepsy in that region or country. However, even so, population-based studies can cover a wider area than clinical sample-based studies and provide a more precise and scientific prevalence of epilepsy in children with CP.

In terms of ethnic group, the prevalence of epilepsy in CP was significantly higher in Black-majority countries (48.6% and 38.1%) than in other-majority countries in both clinical sample-based and population-based studies, which may be associated with poor economics. In the clinical sample-based study, the prevalence of epilepsy was higher in low- and middle-income countries than in high-income countries (47.0% vs. 43.0%). Firstly, due to the low level of parental education, lack of CP-related professional knowledge, poor perinatal and postpartum management, and poor early diagnosis and intervention, CP is generally more severe in motor and cognitive impairment, and the possibility of secondary complications such as epilepsy increases (84–86). Secondly, compared to high-income countries, children from low- and middle-income countries were more often admitted to hospitals for treatment of severe CP, and this segment of the CP population had a high prevalence of epilepsy (87). In contrast, in population-based studies, the prevalence of epilepsy in low- and middle-income countries and high-income countries was very close (30.7% vs. 32.1%), which might be responsible for filling that missing segment of children with mild to moderate CP. The number of articles we included (*n* = 43) and individuals (*n* = 31,098) were highest in countries of predominantly European origin, which may give a more accurate prevalence of epilepsy in this segment of the CP population (46.6% and 31.2%). The low prevalence of epilepsy in Mongolian-majority countries (32.8% and 29.6%) might be because we restricted the search language to English and missed some articles published in non-English languages. Moreover, the number of articles included in this study was generally low (*n* = 8), and there might be some publication bias. The veracity of the results remains to be verified by a large number of original studies.

Based on the time of publication of the article, it was found that the prevalence of epilepsy in CP was low in 1998–2002 (39.9% and 26.4%) compared with other periods, and the prevalence stabilized in 2011–2022, both in clinical sample-based studies and population-based studies. Our interpretation of the results was that it might not be that the prevalence of epilepsy in CP has increased in the last decade. The diagnostic sensitivity or specificity of epilepsy had increased with the updating of epilepsy diagnostic criteria and the promotion of improved assessment techniques, resulting in more children with CP being accurately diagnosed with epilepsy. For example, epilepsy diagnosis in the articles from 1998 to 2002 relied more on the clinical history and symptoms for diagnosis, whereas articles from 2011 to 2022 used clinical symptoms combined with EEG more often. Secondly, it also correlated with the number of articles included. Our inclusion was mainly after 2010 (*n* = 57) and generally less before 2010 (*n* = 15), which might have some publication bias. Regarding gender, the prevalence of CP in males was significantly higher than in females. However, there was no significant difference between groups in the prevalence of epilepsy in the clinic-based and population-based studies, suggesting that gender had a negligible effect on the prevalence of epilepsy.

Several articles suggested that a history of NS was associated with a high risk of epilepsy in CP and was a strong predictor of epilepsy in children with CP. Levene et al. (88) reported that NS adversely affected the developmental progression of CNS and can lead to intrinsic brain lesions that might predispose to cognitive, behavioral, or epileptic problems later in life. It was postulated that NS, especially those starting within the first 72 h, was a significant risk factor for developing epilepsy (31). In addition, Mert et al. (20) showed that children with CP with a history of NS were 3.3 times more likely to have a poor prognosis for epilepsy than those without a history of neonatal seizures. Therefore, a history of NS in CP was a significant related factor for the development and poor prognosis of epilepsy.

Our study found a high prevalence of epilepsy in CP with a family history of epilepsy (89.2%) compared to those without a family history of epilepsy (49.0%). Mert et al. (20) showed that the prevalence of epilepsy in children with CP with a family history of epilepsy was 5.5 times higher than that in children without a family history of epilepsy. Therefore, it can be hypothesized that genetic factors play an essential role in the pathogenesis of epilepsy development and can be a significant predictor of seizures in children with CP.

It is well known that intellectual disability and CP often occur together (89). The prevalence of epilepsy in CP with cognitive/intellectual disability increased with the degree of intellectual disability, probably because CP with epilepsy have more severe brain injury. Thus the coexistence of intellectual disability and epilepsy was high, and each seizure might aggravate the brain injury in children with CP and have some effect on their intelligence (12, 47). According to the study conducted by Karatoprak et al. (31), the prevalence of epilepsy in children with severe intellectual disabilities was found to be 8.9 times higher compared to children with average intelligence. El-tallowy et al. (47) showed that children with CP might suffer from extensive brain injury to various parts of the brain, such as brain gray matter, deep brain white matter, and the central nucleus. Therefore, they were vulnerable to intellectual disability and epilepsy. Cognitive/intellectual disability was uncommon in diplegia because it generally did not damage brain gray matter. In contrast, epilepsy and cognitive/intellectual disability were more common and severe in quadriplegia because of extensive brain injury (36). Although cognitive/intellectual disability is more common in children with CP with severe brain injury, and the possibility of seizures is higher, some children with severe brain injury have normal intelligence and do not often accompany by epilepsy. This may be related to the site of injury. For example, more than half of the intelligence of dyskinetic CP with deep gray matter injury is normal and may not be accompanied by epilepsy (90).

Further study of the possible risk of epilepsy development and its relationship with EEG and cranial imaging might help to find the risk factors associated with epilepsy affecting CP (16). In this study, the cranial imaging applied in the included articles included CT and MRI. Cranial MRI was the most recommended neuroimaging modality. Widely-adopted MRICS could be used for brain abnormalities—malformations, predominantly white matter injuries, mostly gray matter injuries, and miscellaneous and normal imaging. It helps to provide further insights into the nature of the abnormalities to aid the diagnosis, treatment planning, and monitoring of patients with CP. The epilepsy diagnosis was based on family and personal history and symptoms, and EEG was a gold standard for diagnosis (91). Hanci et al. (32) showed that widespread or focal epileptiform abnormalities might be essential in developing CP epilepsy. The data showed that the likelihood of co-morbid epilepsy would be higher in CP with the presence of EEG abnormalities (60.8%) and cranial imaging abnormalities (57.8%) and was statistically different between groups of children with CP without epilepsy (*P* < 0.05). That suggests the importance of early and effective EEG and cranial imaging combined with clinical history for detecting epilepsy in CP.

Many studies have shown that gray matter injury was a significant cause of seizures compared to white matter injury. For example, temporal and frontal lobe injury was highly epileptogenic (7, 46, 73). This study also validated the higher odds of seizures in CP with predominantly gray matter injury (55.9%) compared to predominantly white matter injuries (36.5%) in the present study. However, if the brain white matter was affected or the damage was limited to the basal ganglia and thalamus, the likelihood of epilepsy became low (33). In addition, this study showed a 52.8% prevalence of epilepsy in brain malformations. Carlsson et al. (46) found an increased frequency of seizures in young people with CP with CNS infections and malformations with the injury to the brain gyrus.

Due to the heterogeneity of symptoms and complications of CP, there was no clear global consensus on the type of CP (1, 92). Therefore, in this study, we analyzed CP according to its clinical type (spastic, dyskinetic, ataxic, and mixed) and topographical type (hemiplegia, diplegia, and quadriplegia) based on the typing characteristics of the included articles to explore the differences. This study showed that clinical type was not responsible for the prevalence of epilepsy in CP, but the topographical type mainly affected seizures in children with CP (*P* < 0.001). This study showed that quadriplegia was the leading cause of seizures affecting children with CP (61.8%), followed by hemiplegia (40.2%) and diplegia (26.3%). Because quadriplegia was characterized by extensive brain injury or brain softening secondary to ischemic-hypoxic events with a higher degree of injury and a high prevalence of epilepsy compared to hemiplegia and diplegia (33). Most hemiplegia will have focal cortical injury or infarction due to perinatal arterial ischemic infarction and a higher prevalence of epilepsy. In contrast, diplegic injury tended to be predominantly periventricular leukomalacia, not involving cortical gray matter, and had a lower prevalence of epilepsy (13, 93, 94).

In terms of clinical type, our study included 12,711 young people with CP, 82.5% of them were spastic CP, the most clinical type of CP. And 42.7% of them had epilepsy, the epilepsy prevalence rate was lower than that of the dyskinetic and mixed types. The reason for this was that in addition to the spastic CP only described in some articles, we also included the description of spastic diplegia, spastic hemiplegia, and spastic quadriplegia in a unified category as spastic CP, with a higher proportion of young people with spastic diplegia (34%), resulting in a lower overall prevalence of epilepsy in spastic CP than that in the other clinical types. In this study, children with dyskinetic type had the highest prevalence in studies with clinical as a typing criterion. However, children with simple dyskinetic CP with only basal ganglia lesions were less likely to have epilepsy. There are some reasons for that. First, the dyskinetic type had a higher incidence of neonatal seizures (53). Second, brain injuries in the temporal and frontal lobes were high risk of suffering seizure (53). Third, the more dif ficulty distinguishing between complex partial seizures and the dyskinetic pattern caused an overdiagnosis of epilepsy (53). Fourth, the number of dyskinetic-type individuals in each article was generally few, which had a certain bias from the results. In addition, the ataxic type had the lowest prevalence of epilepsy, but even then, there was a significant incidence of epilepsy (39.1%). We interpreted the reason for this to be that although the prevalence of epilepsy due to cerebellar injury was less likely, various high-risk factor injury were not limited to the cerebellum because the diagnosis of CP was based on clinical symptoms (14). The type of diagnosis of CP might be limited only to the most significant clinical symptoms. Therefore, our interpretation of the results preferred that the severity of the injury and the area of brain injury in CP may be the main factors affecting the onset of epilepsy rather than clinical typing.

The high prevalence of epilepsy in CP might be due to common etiology and risk factors. It was unclear why the same lesions cause CP with or without epilepsy, and we speculated that it might be due to genetic susceptibility. In this study, the prevalence of epilepsy in children with CP born prematurely with low birthweight was lower than that in children born at term and average weight, suggesting that preterm birth and low birth weight were not significant risk factors contributing to seizures in children with CP. Prematurity is caused predominantly periventricular leukomalacia the leading cause of diplegia in preterm infants. In contrast, due to brain gray matter injury, CP was more common in full-term infants (12, 16, 95, 96). Since epilepsy was uncommon in children with spastic diplegia and white matter injury, epilepsy was rarely seen in preterm infants (95).

## 5. Advantages of this study

The advantages of this study include several key points. Firstly, it represents the first and most comprehensive systematic review and meta-analysis conducted on the prevalence of epilepsy in individuals with CP. This study encompasses a larger number of included articles and individuals, resulting in more robust prevalence estimates and increased confidence in the findings. Secondly, a meticulous approach was taken to categorize the articles into clinical sample-based and population-based studies based on the source of samples. This division allows for a more refined analysis and comparison of prevalence rates. Additionally, subgroup analyses were conducted considering factors such as gender, ethnic group, and national income level. This approach helps to mitigate the heterogeneity among studies and provides a more scientifically sound method to assess the prevalence of epilepsy in different subgroups. Thirdly, the study delves into a comprehensive analysis of various confounding factors associated with CP, such as gestational age, birth weight, history of NE, cognitive/intellectual status, and the specific type of CP. By considering these factors, the prevalence of epilepsy was examined from a more comprehensive perspective. Moreover, potential sources of heterogeneity and factors influencing the prevalence of epilepsy were identified. This aids in better understanding the variations observed in the prevalence estimation and provides insights into the associated factors contributing to the occurrence of epilepsy in individuals with CP. Overall, the advantages of this study lie in its comprehensive nature, meticulous categorization and analysis of factors, and the identification of potential sources of heterogeneity. These aspects contribute to a more thorough understanding of the prevalence of epilepsy in individuals with CP.

## 6. Limitations

There are several limitations to consider in this study. Firstly, we did not conduct an age-based meta-analysis of epilepsy prevalence due to the wide age ranges covered in the included studies, which made age group comparisons unfeasible. However, it is worth noting that previous research has shown a higher occurrence of epilepsy in the early years of individuals with CP, with most seizures occurring within the first year of life (14, 17, 22, 30, 33). Furthermore, some population-based studies on adolescents have an age range of up to 19 years, exceeding the commonly accepted age of 18 (45, 58, 62, 68). This necessitated our decision to divide the age range accordingly. Moreover, the single combined estimate of prevalence should be interpreted with caution due to several limitations. Firstly, heterogeneity exists even in subgroup analyses, which is often difficult to avoid in epidemiological studies (97). Secondly, variations in prevalence between studies may stem from factors such as sample source, different definitions of CP and epilepsy, representativeness of samples, and the specificity of diagnostic tools. Many studies relied on clinical history and symptoms to confirm epilepsy, which can result in both under- and over-diagnosis. Thirdly, the data of some articles are not comprehensive enough, such as the failure to reflect the time range of data collection, diagnostic criteria of epilepsy, age range and female ratio on the Table 1. Some articles were excluded from subgroup analysis due to the lack of CP characteristics data, and the number of articles included in subgroup analysis may have a certain bias on the results. Additionally, the high prevalence of epilepsy in CP observed in clinical sample-based studies may be due to an over-representation of more severe individuals admitted to medical institutions for intellectual disability, epilepsy, motor disorders, and speech disorders. Furthermore, it is essential to note that this study excluded unpublished articles and articles published in non-English languages, which could have resulted in an underestimation or overestimation of the true prevalence of epilepsy in CP. Lastly, differences in the characteristics of the individuals in the study may have influenced the results. While this study provides valuable insights, these limitations should be considered when interpreting the findings.

## 7. Conclusion

In summary, our findings indicate that more than one-third of young people with CP experience epilepsy, with an overall prevalence of 38.0%. Prevalence rates differ based on the study type, with clinic-based studies showing a prevalence of 46.4% and population-based studies showing a prevalence of 31.6%. The source of CP cases in the articles emerged as a significant factor contributing to the heterogeneity in epilepsy prevalence. Our analysis suggests NE, a family history of epilepsy, abnormal EEG findings, abnormal cranial imaging, severe intellectual disability, and quadriplegia may be associated with comorbid epilepsy in CP. Given the high prevalence of epilepsy in CP, it is recommended that children with these related factors be followed up regularly for a long time. Cranial imaging and EEG should be performed on young people with CP suspected of having a risk of seizures, and appropriately use anticonvulsant medication to reduce seizure frequency in individuals with CP. Furthermore, systematic investigations into the prevalence of epilepsy in individuals with CP and exploring factors contributing to seizures may also shed light on potential shared genetic mechanisms, providing new insights into the genetic patterns and possible overlaps between these disorders.

## Data Availability

The original contributions presented in the study are included in the article/**Supplementary Material**, further inquiries can be directed to the corresponding authors.
