# Supplementary material for: Prevalence and related factors of epilepsy in children and adolescents with cerebral palsy: a systematic review and meta-analysis
Source: Front Pediatr. 2023 Jul 28;11:1189648. doi: 10.3389/fped.2023.1189648 (PMC10416728; doi:10.3389/fped.2023.1189648)
Supplement: Supplementary file 1 [file Datasheet1.pdf]

一， Search strategy

1.2.1 Pubmed: 605

#1: "Cerebral Palsy"[Mesh]

#2: ((("Cerebral Pals*"[Title/Abstract]) OR (CP[Title/Abstract])) OR ("Little Disease"[Title/Abstract])) OR ("Spastic Diplegia*"[Title/Abstract])

#3: #1 OR #2

#4: "Epilepsy"[Mesh]

#5: "Seizures"[Mesh]

#6: (((Aura*[Title/Abstract]) OR (Epileps*[Title/Abstract])) OR (Seizure*[Title/Abstract])) OR (Convulsion[Title/Abstract])

#7: #4 OR #5 OR #6

#8: (((prevalence[Title/Abstract]) OR (epidemiology[Title/Abstract])) OR (rate[Title/Abstract]))

#4: #3 AND #7 AND #8

((((("Cerebral Pals*"[Title/Abstract]) OR (CP[Title/Abstract])) OR ("Little Disease"[Title/Abstract])) OR ("Spastic Diplegia*"[Title/Abstract])) AND ((((Aura*[Title/Abstract]) OR (Epileps*[Title/Abstract])) OR (Seizure*[Title/Abstract])) OR (Convulsion[Title/Abstract]))) AND ((((morbidity[Title/Abstract]) OR (epidemiology[Title/Abstract])) OR (rate[Title/Abstract])) OR (prevalence[Title/Abstract]))

1.2.2 Web of science: 904

#1: (((TS=("cerebral pals*")) OR TS=(CP)) OR TS=("Little Disease")) OR TS=("Spastic Diplegia*")

#2: (((TS=(seizure*)) OR TS=(epileps*)) OR TS=(Aura*)) OR TS=(Convulsion)

#3: (((TS=(prevalence)) OR TS=(epidemiology)) OR TS=(rate)) OR TS=(morbidity)

#4: #1 AND #2 AND #3

((((TS=("cerebral pals*")) OR TS=(CP)) OR TS=("Little Disease")) OR TS=("Spastic Diplegia*")) AND ((((TS=(seizure*)) OR TS=(epileps*)) OR TS=(Aura*)) OR TS=(Convulsion)) AND (((TS=(prevalence)) OR TS=(epidemiology)) OR TS=(rate)) OR TS=(morbidity))

1.2.3 Embrace: 1511

#1: 'cerebral palsy'/exp
#2: 'cerebral palsy':ti,ab
#3: cp:ti,ab
#4: 'little disease':ab,ti
#5: 'spastic diplegia*':ab,ti
#6: 'epilepsy'/exp
#7: 'seizures'/exp
#8: epileps*:ab,ti
#9: seizure*:ab,ti
#10: aura*:ab,ti
#11: convulsion:ab,ti
#12: prevalence:ab,ti
#13: epidemiology:ab,ti
#14: rate:ab,ti
#15: #1 OR #2 OR #3 OR #4 OR #5
#16: #6 OR #7 OR #8 OR #9 OR #10
#17: #11 OR #12 OR #13
#18: #15 AND #16 AND #17

二， Remove repetitive literature

A total of 3020 articles were retrieved, and 1379 duplicates were removed by endnote20.

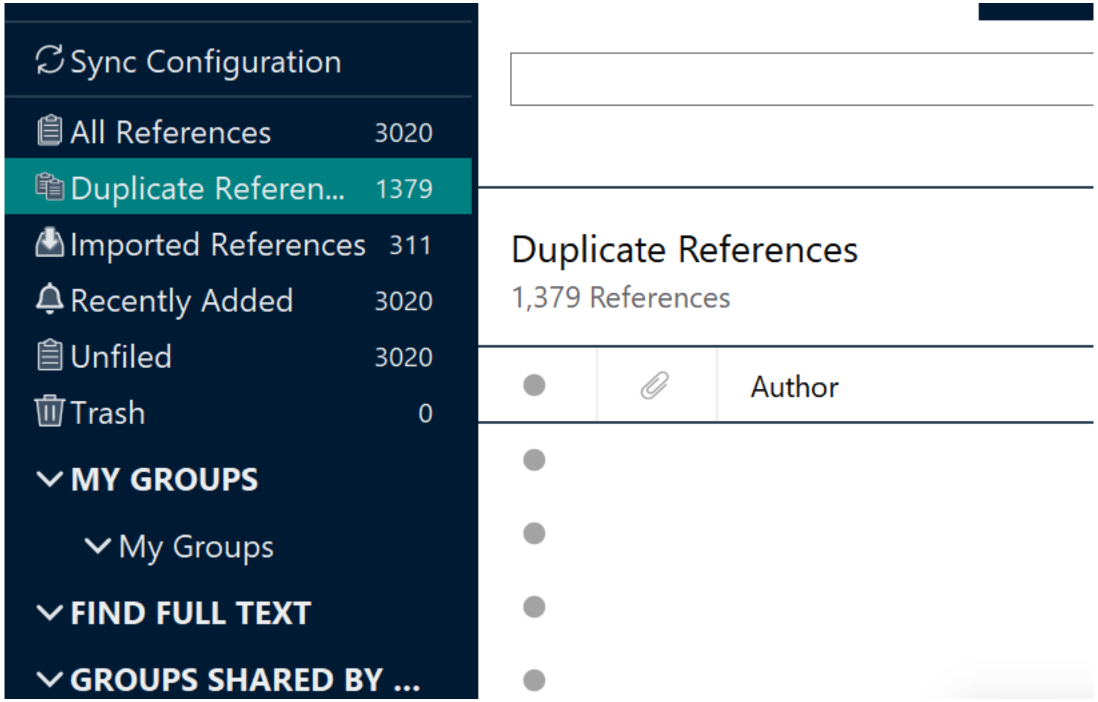

三，screening（after reviewing titles and abstracts）

(a) first screening: 99+30+75=204

(b) secondary screening: 204-135=69

(c) Total=other source(3) + secondary screening(69)=72

NO.	Title（form pubmed）	Years	First auther	Journal	Secondary screening(after reviewing full-text)
1	Pattern and Predictors of Epilepsy among Children with Cerebral Palsy in Jos, Nigeria.	2020	Ejeliogu	West Afr J Med	included
2	Seizure outcome and epilepsy patterns in patients with cerebral palsy.	2019	Tantawi	Seizure	included

3	Long-term prognosis of epilepsy in patients with cerebral palsy.	2019	Tsubouchi	Dev Med Child Neurol	included
4	Progression of motor disability in cerebral palsy: The role of concomitant epilepsy.	2020	Takano	Seizure	Study on a type of CP
5	The epileptology of alternating hemiplegia of childhood.	2019	Uchitel	Neurology	included
6	Epilepsy and drug-resistant epilepsy in children with cerebral palsy: A retrospective observational study.	2020	Hanci	Epilepsy Behav	Study on a type of CP
7	Seizures in Children With Cerebral Palsy and White Matter Injury.	2017	Cooper	Pediatrics	Study on a type of CP
8	Risk factors associated with epilepsy development in children with cerebral palsy	2019	Karatoprak	Childs Nerv Syst	Study on a type of CP
9	Risk factors and prognosis of epilepsy in children with cerebral palsy in north-eastern Poland.	2003	Kułak	Brain Dev	Study on a type of CP
10	High rates of malnutrition and epilepsy: two common comorbidities in children with cerebral palsy.	2019	Aydın	Turk J Med Sci	Study on a type of CP
11	Epilepsy and cerebral palsy: characteristics and trends in children born in 1976-1998.	2012	Sellier	Eur J Paediatr Neurol	included
12	Profile of children with cerebral palsy at a tertiary hospital in eastern Nepal	2022	Chaudhary	BMC Pediatr	included
13	The Role of Prematurity in Patients With Hemiplegic Cerebral Palsy.	2016	Zelnik	J Child Neurol	Study on a type of CP
14	Risk factors for epilepsy in children with cerebral palsy.	2010	Zelnik	Eur J Paediatr Neurol	included
15	Factors affecting epilepsy development and epilepsy prognosis in cerebral palsy.	2011	Mert	Pediatr Neurol	included
16	A survey-based report on the occurrence of cerebral palsy in Urban areas of Karachi.	2020	Rafique	J Pak Med Assoc	included
17	Characteristics and prognosis of epilepsy in children with cerebral palsy	1999	Zafeiriou	J Child Neurol	included
18	Clinical and aetiological aspects of epilepsy in children with cerebral palsy.	2003	Carlsson	Dev Med Child Neurol	included
19	Hemiplegic (unilateral) cerebral palsy in northern Stockholm: clinical assessment, brain imaging, EEG, epilepsy and aetiologic background factors.	2020	Tillberg	BMC Pediatr	Study on a type of CP
20	Improving epilepsy control among children with cerebral palsy in rural Bangladesh: a prospective cohort-based study	2022	Karim	BMJ Open	included
21	[Clinical analysis of 322 cases of non-epileptic cerebral palsy]	2010	Zhu	Zhongguo Dang Dai Er Ke Za Zhi	Non-English articles
22	Epilepsy in children with cerebral palsy.	2001	Aneja	Indian J Pediatr	included
23	Epilepsy in children with cerebral palsy.	2001	Bruck	Arq Neuropsiquiatr	included
24	Epileptic and non-epileptic cerebral palsy: EEG and cranial imaging findings.	2002	Senbil	Brain Dev	included

25	Epilepsy in children with cerebral palsy.	2003	Singhi	J Child Neurol	included
26	Epilepsy in children with cerebral palsy.	1998	Kwong	Pediatr Neurol	included
27	Epilepsy in patients with cerebral palsy.	1997	Lagunju	Dev Med Child Neurol	Abstracts
28	Epilepsy in children with cerebral palsy.	2003	Gururaj	Seizure	Study on a type of CP
29	Epilepsy in patients with cerebral palsy--analysis of frequency and clinical prognosis	2003	Sugiura	No To Hattatsu	Non-English articles
30	Profile of associated impairments at age 5 years in Australia by cerebral palsy subtype and Gross Motor Function Classification System level for birth years 1996 to 2005.	2016	Delacy	Dev Med Child Neurol	included
31	[Epilepsy in children with cerebral palsy]	2006	Peduzzi	Rev Med Liege	Abstracts
32	Hemiplegic cerebral palsy. Aetiology and outcome.	1988	Uvebrant	Acta Paediatr Scand Suppl	Study on a type of CP
33	Epilepsy in Saudi children with cerebral palsy.	2001	Al-Sulaiman	Saudi Med J	Abstracts
34	Epilepsy in children with congenital hemiplegia: correlation between clinical, EEG and neuroimaging findings.	2002	Panteliadis	Epileptic Disord	Abstracts
35	Comorbidities in patients with cerebral palsy and their relationship with neurologic subtypes and Gross Motor Function Classification System levels	2010	Hou	Zhonghua Er Ke Za Zhi	Non-English articles
36	Control of epilepsy in children and adolescents suffering from cerebral palsy, with respect to their etiology and cerebral lesion background	2006	Rolón-Lacarr iere	Rev Neurol	Abstracts
37	Australian Cerebral Palsy Child Study: protocol of a prospective population based study of motor and brain development of preschool aged children with cerebral palsy.	2013	Boyd	BMC Neurol	no data
38	Epileptic seizures in children and youth with cerebral palsy	2000	Mieszczanek	Neurol Neurochir Pol	Abstracts
39	Congenital and acquired hemiplegia in children: epileptic crisis and eletrencephalografic signs	2005	Silva	Acta Cir Bras	Abstracts
40	Cerebral palsy and epilepsy in children	2005	Svraka	Med Arh	No full text
41	Epilepsy in children with cerebral palsy.	2004	Rahman	Mymensingh Med J、	No full text
42	Association of cerebral palsy with epilepsy	1997	Kaushik	J Indian Med Assoc	Abstracts
43	Comparative analysis of prognostic value of epileptic encephalopathies and symptomatic epilepsy in children with cerebral palsy	2005	Kakushadze	Georgian Med News	No full text

44	Epilepsy in different types of cerebral palsy	2003	Diaconu	Rev Med Chir Soc Med Nat Ias	Abstracts
45	Seizures in patients with cerebral palsy	1990	Hosking	Dev Med Child Neurol	no data
46	[Cerebral palsy in childhood: 250 cases report]	2002	Bringas-Grande	Rev Neurol	No full text
47	Epidemiological study of cerebral palsy in Shiga Prefecture, Japan, during 1977-2000. Part 3: clinical features of cerebral palsy at six years of age	2009	Suzuki	No To Hattatsu	No full text
48	A clinical study of cerebral palsy in Shiga; 1977-1986--II. Severity of the disability and complications in various types of cerebral palsy	1999	Suzuki	No To Hattatsu	Abstracts
49	An epileptic syndrome in infantile cerebral palsy	1997	Sumerkina	Zh Nevrol Psikhiatr Im S S Korsakova	Abstracts
50	Epilepsy in Nigerian children with cerebral palsy	2007	Lagunju	Dev Med Child Neurol	included
51	Cerebral palsy following term newborn encephalopathy: a population-based study.	2005	Badawi	Dev Med Child Neurol	Not interest of epilepsy in CP
52	Clinical features and etiology of epilepsy in children with cerebral palsy	2005	Wen	Zhonghua Er Ke Za Zhi	Non-English articles
53	Antecedents and neuroimaging patterns in cerebral palsy with epilepsy and cognitive impairment: a population-based study in children born at term.	2017	Ahlin	Acta Obstet Gynecol Scand	no data
54	A prospective comparative study of the aetiology of cerebral palsy and epilepsy in a one-year birth cohort from Northern Finland.	1986	Rantakallio	Acta Paediatr Scand	no data
55	The design of a population register on cerebral palsy: its application and analysis in Andorra and Navarre	2018	Avellanet	Rev Neurol	No full text
56	The usefulness of MRI Classification System (MRICS) in a cerebral palsy cohort	2020	Nagy	Acta Paediatr	no data
57	Prevalence of Intellectual Disabilities and Epilepsy in Different Forms of Spastic Cerebral Palsy in Adults.	2017	Vukojević	Psychiatr Danub	No full text
58	Association of cerebral palsy with other disabilities in children with perinatal arterial ischemic stroke.	2007	Golomb	Pediatr Neurol	Study on a type of CP
59	Prevalence of cerebral palsy, co-occurring autism spectrum disorders, and motor functioning - Autism and Developmental Disabilities Monitoring Network, USA, 2008.	2014	Christensen	Dev Med Child Neurol	included
60	Cerebral palsy in Moldova: subtypes, severity and associated impairments.	2018	Bufteac	BMC Pediatr	included
61	Prevalence and demographic characteristics of comorbid epilepsy in children and adolescents with cerebral	2019	Chiang	Childs Nerv Syst	included

	palsy: a nationwide population-based study.				
62	Prevalence and functioning of children with cerebral palsy in four areas of the United States in 2006: a report from the Autism and Developmental Disabilities Monitoring Network.	2011	Kirby	Res Dev Disabil	included
63	Epidemiological, clinical, and treatment-related features of children with cerebral palsy in Cameroon: A hospital-based study	2022	Mangamba	Arch Pediatr	included
64	Prevalence and factors associated with sleep disorders among children with cerebral palsy in Uganda; a cross-sectional study	2018	Munyumu	BMC Pediatr	Study on a type of CP
65	Comorbidities and clinical determinants of outcome in children with spastic quadriplegic cerebral palsy.	2008	Venkateswaran	Dev Med Child Neurol	Study on a type of CP
66	Cerebral palsy and additional handicaps in a 1-year birth cohort from northern Finland--a prospective follow-up study to the age of 14 years.	1985	Wendt	Ann Clin Res	Study on a type of CP
67	Epidemiology of cerebral palsy in Sumba Island, Indonesia.	2020	Jahan	Dev Med Child Neurol	included
68	Epilepsy in children with cerebral palsy: a data linkage study.	2022	Szpindel	Dev Med Child Neurol	included
69	Factors contributing to the longitudinal development of social participation in individuals with cerebral palsy.	2016	Tan	Res Dev Disabil	included
70	Trends in prevalence and characteristics of cerebral palsy among Icelandic children born 1990 to 2003.	2009	Solveig	Dev Med Child Neurol	included
71	Survey on children with cerebral palsy in Tochigi Prefecture, Japan.	2021	Hirokazu	Pediatr Int	included
72	Predictors for early diagnosis of cerebral palsy from national registry data.	2015	Jakob	Dev Med Child Neurol	included
73	Prevalence of cerebral palsy among ten-year-old children in metropolitan Atlanta, 1985 through 1987.	1993	Murphy	J Pediatr	no data
74	Evaluation of the relationship between cranial magnetic resonance imaging findings and clinical status in children with cerebral palsy.	2021	Şık	Turk J Med Sci	included
75	Cerebral palsy in children in Kampala, Uganda: clinical subtypes, motor function and co-morbidities.	2015	Kakooza-Mwesige	BMC Res Notes	included
76	Cerebral palsy in Dakar	2002	Ndiaye	Dakar Med	No full text
77	Cerebral palsy and epilepsy: a health informatics approach	2022	Chin	Dev Med Child Neurol	Review articles
78	Incidence of neonatal seizures, perinatal risk factors for epilepsy and mortality after neonatal seizures in the province of Parma, Italy	2018	Pisani	Epilepsia	Study on a type of CP

79	Trends in prevalence and characteristics of post-neonatal cerebral palsy cases: a European registry-based study	2013	Germany	Res Dev Disabil	Included
80	Cerebral palsy in preterm infants	2016	Drljan	Vojnosanit Pregl	no data
81	Cerebral palsy in Norway: prevalence, subtypes and severity	2008	Andersen	Eur J Paediatr Neurol	included
82	Comorbidities in cerebral palsy: a patient registry study.	2020	Hollung	Dev Med Child Neurol	included
83	Cerebral palsy prevalence, subtypes, and associated impairments: a population-based comparison study of adults and children	2019	Jonsson	Dev Med Child Neurol	included
84	Cerebral palsy in southern Sweden I. Prevalence and clinical features	2001	Nordmark	Acta Paediatr	included
85	Cerebral Palsy and Epilepsy in Children: Clinical Perspectives on a Common Comorbidity	2020	Pavone	Children (Basel)	included
86	The Profile of Epilepsy and its characteristics in Children with Cerebral Palsy.	2022	Saini	Seizure	included
87	Cerebral palsy in children: subtypes, motor function and associated impairments in Addis Ababa, Ethiopia.	2021	Tsige	BMC Pediatr	included
88	Congenital anomalies in children with cerebral palsy in rural Bangladesh.	2020	Ellen	Dev Med Child Neurol	no data
89	Pattern of comorbidities in school-aged children with cerebral palsy in Cross River State, Nigeria.	2021	Duke	BMC Pediatr	included
90	Epidemiology of cerebral palsy in Bangladesh: a population-based surveillance study.	2019	Khandaker	Dev Med Child Neurol	included
91	One-third of school-aged children with cerebral palsy have neuropsychiatric impairments in a population-based study	2019	Påhlman	Acta Paediatr	included
92	Clinical Spectrum, Comorbidities, and Risk Factor Profile of Cerebral Palsy Children: A Prospective Study.	2017	Minocha	J Pediatr Neurosci	included
93	Epilepsy syndromes in cerebral palsy: varied, evolving and mostly self-limited.	2022	Cooper	Brain	Study on a type of CP
94	Epileptic and cognitive changes in children with cerebral palsy: an Egyptian study	2014	El-Tallawy	Neuropsychiatr Dis Treat	included
95	Risk Factors of Intractable Epilepsy in Children with Cerebral Palsy	2021	Maksoud	Iran J Child Neurol	Study on a type of CP
96	Evaluation of Risk Factors for Epilepsy in Pediatric Patients with Cerebral Palsy	2020	Sadowska	Brain Sci	included
97	Epilesy in Saudi children with cerebral palsy	2001	Al-Sulaiman	Neurosciences (Riyadh)	Abstracts
98	Cerebral Palsy in North Indian Children: Clinico-etiological Profile and Comorbidities.	2019	Bhati	J Pediatr Neurosci	Study on a type of CP
99	Clinical Spectrum of Cerebral Palsy and Associated Disability in South Egypt: A Local Survey Study.	2017	Abas	Open Access Maced J Med Sci	included

NO.	Title（form web of science, after remove duplicate articles with pubmed）	Years	First auther	Secondary screening(after revie
1	Sleep disorders and associated factors in children with cerebral palsy	2021	Patery	Study on a type of CP
2	Determinants of Hearing Loss in Children with Cerebral Palsy in Kano, Nigeria	2021	Jibril	included
3	Relationship of Environmental Factors With Social Participation of Children With Cerebral Palsy Spastic Diplegia: A Preliminary Study	2021	Gharib	Study on a type of CP
4	Developmental anomalies and associated impairments in Saudi children with cerebral palsy: a registry-based, multicenter study	2021	Al-Garni	included
5	Study on Etiological Factors, Clinical Pattern and Comorbidities in Cerebral Palsy Children of North Bihar Region	2020	Kumar	Abstracts
6	ASSOCIATION OF RISK FACTORS OF CEREBRAL PALSY - A MATCHED CASE CONTROL STUDY	2020	Hemachithra	Abstracts
7	Impairments, functional limitations, and access to services and education for children with cerebral palsy in Uganda: a population-based study	2020	Andrews	included
8	Clinical features and aetiology of cerebral palsy in children from Cross River State, Nigeria	2020	Duke	no data
9	EPIDEMIOLOGY, COST AND ECONOMIC IMPACT OF CEREBRAL PALSY IN HUNGARY	2019	Fejes	no data
10	Determining Risk Factors of Epilepsy in Children with Cerebral Palsy: A Retrospective Study	2018	Gurkan	Not interest of epilepsy in CP
11	Decreasing prevalence and severity of cerebral palsy in Norway among children born 1999 to 2010 concomitant with improvements in perinatal health	2018	Hollung	included
12	Demographic and Clinical Characteristics of Patients with Cerebral Palsy	2018	Sucuoglu	included
13	Congenital anomalies and the severity of impairments for cerebral palsy	2017	Jystad	Study on a type of CP
14	Frequency of Various Types of Cerebral Palsy amongst the Admitted Children at a Tertiary Care Hospital and Retrospective Etiologic Analysis on the Basis of History, Examination and Laboratory Support	2017	Ali	no data
15	Temporal trends in cerebral palsy by impairment severity and birth gestation	2016	Reid	no data
16	Obstructive sleep apnea in children with cerebral palsy and epilepsy	2016	Garcia	included
17	Pediatric Cerebral Palsy in Botswana: Etiology, Outcomes, and Comorbidities	2016	Bearden	included

**18**	**Tertiary paediatric hospital admissions in children and young people with cerebral palsy**	**2015**	**Meehan**	included
**19**	**Demographic and Clinical Findings of Cerebral Palsy Patients in Istanbul: A Multicenter Study**	**2014**	**Yalcinkaya**	included
**20**	**Changes in the Clinical Spectrum of Cerebral Palsy over Two Decades in North India-An Analysis of 1212 Cases**	**2013**	**Singhi**	included
**21**	**Magnetic resonance imaging, risk factors and co-morbidities in children with cerebral palsy**	**2011**	**Prasad**	included
**22**	**Function and neuroimaging in cerebral palsy: a population-based study**	**2011**	**Himmelmann**	included
**23**	TREATMENT OF EPILEPSY IN CHILDREN WITH DEVELOPMENTAL DISABILITIES	**2010**	**Depositario-Cabacar**	no data
**24**	**Demographic Data and Clinical Characteristics of 202 Cerebral Palsy Cases**	**2009**	**Eriman**	Not interest of epilepsy in CP
**25**	**Dyskinetic cerebral palsy in Europe: trends in prevalence and severity**	**2009**	**Himmelmann**	Study on a type of CP
**26**	**The epidemiology of cerebral palsy: Incidence, impairments and risk factors**	**2006**	**Odding**	Not interest of epilepsy in CP
**27**	**Neuroimpairments, activity limitations, and participation restrictions in children with cerebral palsy**	**2002**	**Beckung**	included
**28**	**The prevalence of epilepsy and other seizure disorders in an Arab population: a community-based study**	**2001**	**Al Rajeh**	Not interest of epilepsy in CP
**29**	**Cerebral palsy in Northern Ireland: 1981-93**	**2001**	**Parkes**	included
**30**	**The changing panorama of cerebral palsy in Sweden .7. Prevalence and origin in the birth year period 1987-90**	**1996**	**Hagberg**	Not interest of epilepsy in CP

NO.	Title（form embase, after remove duplicate articles with pubmed and web of science）	years	First auther	Secondary screening(after reviewing full-text)
**1**	**Cerebral palsy in Switzerland - insights from a national clinical registry and cohort study**	**2022**	**Hunziker**	Abstracts
**2**	**Chronic pain among children with cerebral palsy attending a Ugandan tertiary hospital: a cross-sectional study**	**2021**	**Bambi**	included
**3**	**Cohort profile: The Swiss Cerebral Palsy Registry (Swiss-CP-Reg) cohort study**	**2021**	**Belle**	Not interest of epilepsy in CP
**4**	**Health Conditions in Adults With Cerebral Palsy: The Association With CP Subtype and Severity of Impairments**	**2021**	**Jonsson**	Not interest of epilepsy in CP

**5**	**Specifications of epilepsy and cerebral palsy in children**	**2021**	**Sabirov**	Abstracts
**6**	**Survey of Brazilian children with cerebral palsy: A retrospective study**	**2021**	**Klettenberg**	Abstracts
**7**	**Epidemiology of fractures in children with cerebral palsy in Sweden: A population-based registry study**	**2021**	**Linton**	**included**
**8**	**Age of diagnosis, fidelity and acceptability of an early diagnosis clinic for cerebral palsy: Single site implementation study**	**2021**	**Te Velde**	no data
**9**	**What is genetic about cerebral palsy?**	**2021**	**Twin**	Study on a type of CP
**10**	**Comparison of clinical characteristics and neuroimaging of cerebral palsy with and without epilepsy in children**	**2021**	**Yulianti**	Study on a type of CP
**11**	**Epilepsy and drug-resistant epilepsy in children with cerebral palsy: A retrospective observational study**	**2020**	**Hanci**	Duplicates further identified
**12**	**Pattern and Predictors of Epilepsy among Children with Cerebral Palsy in Jos, Nigeria**	**2020**	**Ejeliogu**	no data
**13**	**Epidemiology of epilepsy in children with cerebral palsy in Southern Nigeria**	**2020**	**Duke**	no data
**14**	**Clinical features and aetiology of cerebral palsy in children from Cross River State, Nigeria**	**2020**	**Duke**	Duplicates further identified
**15**	**Epidemiology of Cerebral Palsy in Adulthood: A Systematic Review and Meta-analysis of the Most Frequently Studied Outcomes**	**2020**	**van Gorp**	Abstract
**16**	**Characteristics of patients with epilepsy and various forms of cerebral palsy**	**2020**	**Magalov**	Abstracts
**17**	**Cerebral palsy: Current opinions on definition, epidemiology, risk factors, classification and treatment options**	**2020**	**Sadowska**	Study on a type of CP
**18**	**The prevalence of malnutrition and the nutritional status in children with cerebral palsy and its causes in madinah maternity and children hospital.**	**2020**	**Aal-Blowi**	included
**19**	**Children with dyskinetic cerebral palsy are severely affected as compared to bilateral spastic cerebral palsy**	**2020**	**Préel**	Study on a type of CP
**20**	**A report on characteristics of cerebral palsy from Henan cerebral palsy register and rehabilitation management system**	**2019**	**Zhu**	Abstracts
**21**	**Nepal cerebral palsy register (NCPR): Towards developing a platform for national cerebral palsy (CP) register and population-based surveillance of children with CP in Nepal**	**2019**	**Muhit**	Abstracts
**22**	**Post-neonatal cerebral palsy 1995-2012: Findings from the Australian Cerebral Palsy Register**	**2019**	**Waight**	no data

**23**	**Cerebral palsy register of Southern Switzerland**	**2019**	**Ruben**	No full text
**24**	**Epilepsy in cerebral palsy**	**2019**	**Magalov**	Not interest of epilepsy in CP
**25**	**Prevalence of chronic pain and associated factors among children with cerebral palsy attending the child neurology and rehabilitation clinics at Mulago National Referral Hospital, Uganda**	**2019**	**Bambi**	Study on a type of CP
**26**	**Children with epilepsy: More than just seizures**	**2019**	**Ladapo**	Study on a type of CP
**27**	**Cerebral palsy in Moldova: Subtypes, severity and associated impairments 11 Medical and Health Sciences 1117 Public Health and Health Services 11 Medical and Health Sciences 1114 Paediatrics and Reproductive Medicine**	**2019**	**Gincota**	Not interest of epilepsy in CP
**28**	**The design of a population register on cerebral palsy: Its application and analysis in Andorra and navarre**	**2018**	**Avellanet**	No full text
**29**	**Intellectual disability in cerebral palsy: a population-based retrospective study**	**2018**	**Reid**	**included**
**30**	**Children with cerebral palsy from the Portuguese National Register, not born in Portugal**	**2018**	**Virella**	Not interest of epilepsy in CP
**31**	**Epidemiology of cerebral palsy in Bangladesh: Findings from the first general population-based CP register in a low and middle-income country**	**2018**	**Khandaker**	Not interest of epilepsy in CP
**32**	**Prevalence of cerebral palsy in Uganda: a population-based study**	**2018**	**Kakooza-Mwesige**	**included**
**33**	**Features of epilepsy and cerebral palsy in children in Uzbekistan**	**2017**	**Tolibov**	Abstracts
**34**	**Associated impairments among children with cerebral palsy in rural Bangladesh: Findings from the Bangladesh Cerebral Palsy Register (BCPR)**	**2017**	**Khandaker**	Not interest of epilepsy in CP
**35**	**Prevalence and characteristics of autism spectrum disorders in children with cerebral palsy**	**2017**	**Delobel-Ayoub**	**included**
**36**	**Risk factors for epilepsy in cases with cerebral palsy: A retrospective study**	**2017**	**Gürkan**	Abstracts
**37**	**Epilepsy in children with cerebral palsy as observed in Nepal**	**2017**	**Thapa**	Abstracts
**38**	**Report from the Austrlian cerebral palsy register (ACPR): Birth prevalence of cp is declining**	**2017**	**Smithers-Sheedy**	No full text
**39**	**Prevalence and characteristics of children with autism spectrum disorders among children with cerebral palsy**	**2016**	**Arnaud**	Not interest of epilepsy in CP
**40**	**Changing trends in cerebral palsy in Victoria, Australia, 1983-2009**	**2016**	**Reid**	Not interest of epilepsy in CP

**41**	**Bangladesh Cerebral Palsy Register (BCPR): Developing a cerebral palsy (CP) register and surveillance of children with CP in a typical lowand middle-income country**	**2016**	**Khandaker**	no data
**42**	**Cerebral palsy in Nigerian children: Profile and impact on educational opportunities**	**2016**	**Lagunju**	Abstracts
**43**	**Time trends in clinical characteristics and diagnostic measures of children with CP in Norway**	**2016**	**Hollung**	no data
**44**	**The epidemiology of intellectual disabilities in cerebral palsy**	**2016**	**Reid**	no data
**45**	**Trends in cerebral palsy in Saskatoon, Saskatchewan in the last four decades**	**2016**	**Sawicka**	Abstracts
**46**	**Trends in cerebral palsy in Saskatoon, Canada in the last four decades**	**2016**	**Sawicka**	no data
**47**	**Gestation-specific trends in cerebral palsy prevalence and impairment severity in Victoria, Australia, 1983-2009**	**2016**	**Reid**	No full text
**48**	**Facilitators and barriers to participation diversity of adults aged 40 to 50 years with cerebral palsy**	**2016**	**Johnston**	No full text
**49**	**Profile of associated impairments at age 5 years in Australia by cerebral palsy subtype and Gross Motor Function Classification System level for birth years 1996 to 2005**	**2016**	**Delacy**	Duplicates further identified
**50**	**Cerebral palsy in children in Botswana: Outcomes and comorbidities**	**2015**	**Baranov**	Not interest of epilepsy in CP
**51**	**Clinical parameters in children with cerebral palsy and epilepsy**	**2015**	**Cerovac**	no data
**52**	**Co-occurring autism spectrum disorder, intellectual disability, and epilepsy among children with cerebral palsy**	**2014**	**Christensen**	No full text
**53**	**Clinical-epidemiological characterization in pediatric patients with cerebral palsy**	**2014**	**Solorio**	Abstracts
**54**	**Cerebral palsy in Al-Quseir City, Egypt: Prevalence, subtypes, and risk factors**	**2014**	**El-Tallawy**	only 46,Too few samples
**55**	**Children with dyskinetic cerebral palsy born in 2001-2004, from the Portuguese national surveillance**	**2014**	**Virella**	No full text
**56**	**Cerebral palsy in Australia, birth years 1993-2006: Findings from the Australian Cerebral Palsy Register**	**2014**	**Gibson**	Not interest of epilepsy in CP
**57**	**Relationship between gross motor function and associated impairments in children with cerebral palsy in Queensland (birth years 1996-2005)**	**2014**	**Delacy**	No full text
**58**	**Clinical spectrum of cerebral palsy in South Jordan: Analysis of 122 cases**	**2013**	**Nafi**	Abstracts
**59**	**Queensland Cerebral Palsy Register-successful consent-based ascertainment of a 10-year cohort**	**2013**	**Delacy**	No full text
**60**	**Demographic data and clinical characteristics of 132 cases of cerebral palsy**	**2013**	**Karao[illegible]lan**	included

**61**	**Epilepsy in children with cerebral palsy**	**2013**	**Bildstein**	no data
**62**	**Epilepsy in Nigerian children with cerebral palsy in Enugu**	**2013**	**Aronu**	Duplicates further identified
**63**	**Cerebral palsy: A senegalese cohort**	**2012**	**Ndiaye**	Non-English articles
**64**	**Demographic, etiological and clinical characteristics of our cerebral palsy cases**	**2012**	**Aksoy**	Abstracts
**65**	**Epilepsy in patients with cerebral palsy is not always lifelong condition**	**2012**	**Ignjatovic**	Not interest of epilepsy in CP
**66**	**Sociocultural issues and causes of cerebral palsy in Port Harcourt, Nigeria**	**2012**	**Frank-Briggs**	included
**67**	**Frequency and types of epilepsy in children with cerebral palsy (CP)**	**2011**	**Saeed**	Abstracts
**68**	**Findings from the inaugural Australian Cerebral Palsy Register (ACPR) report**	**2010**	**Mcintyre**	no data
**69**	**Prevalence and characteristics of cerebral palsy in four areas of the United States in 2006: An update from the autism and developmental disabilities monitoring network**	**2010**	**Kirby**	Duplicates further identified
**70**	**the victorian cerebral palsy register: Learning more about cerebral palsy**	**2010**	**Reddihough**	Not interest of epilepsy in CP
**71**	**Risk factors for epilepsy in children with cerebral palsy**	**2010**	**Zelnik**	Duplicates further identified
**72**	**Measuring health conditions of young children with cerebral palsy**	**2009**	**Bartlett**	no data
**73**	**Pervasive developmental disorders in individuals with cerebral palsy**	**2009**		Duplicates further identified
**74**	**High risk factors of cerebral palsy and clinical characteristics of neurological dysfunction in children: Analysis of 265 cases**	**2004**	**Wang**	Not interest of epilepsy in CP
**75**	**Incidence of epilepsy among patients with cerebral palsy (CP) in Yayasan pemeliharaan anak cacat (YPAC) – Medan**	**2002**	**Sianturi**	included

**NO.**	**Title（form other means）**	**Years**	**First auther**	**Secondary screening(after reviewing full-text)**
1	Cerebral palsy	1998	Singhi	No full text

2	Cerebral palsy: not always what it seems	2001	R Gupta	no data
3	Risk factors for epilepsy in children with cerebral palsy	2010	Zelnik	Duplicates further identified
4	Risk factors and prognosis of epilepsy in children with cerebral palsy in north-eastern Poland	2003	Wojciech Kul⁄ak	Not interest of epilepsy in CP
5	Epilepsy in cerebral palsy	2001	Wallace	Not interest of epilepsy in CP
6	Practice parameter: diagnostic assessment of the child with cerebral palsy: report of the Quality Standards Subcommittee of the American Academy of Neurology and the Practice Committee of the Child Neurology Society	2004	Ashwal	Not interest of epilepsy in CP
7	Clinical and aetiological aspects of epilepsy in children with cerebral palsy	2003	Carlsson	Duplicates further identified
8	The misdiagnosis of epilepsy in children admitted to a tertiary epilepsy centre with paroxysmal events	2006	Uldall	Not interest of epilepsy in CP
9	Data and Statistics for Cerebral Palsy	2021		No full text
10	Epilepsy in children with cerebral palsy	2003	Singh	Duplicates further identified
11	Prevalence of the epilepsies in children and adolescents	1989	Cowan	Not interest of epilepsy in CP
12	Epilepsy in brain-injured children	1990	Aicardi	Not interest of epilepsy in CP
13	Nature and prognosis of seizures in patients with cerebral palsy	1990	Aksu	no data
14	Ingram TTS: Pediatric Aspects of Cerebral Palsy	1962		No full text
15	Hemiplegic cerebral palsy. Aetiology and outcome	1988	Uvebrant	Study on a type of CP
16	Active epilepsy in mentally retarded children. I. Prevalence and additional neuro-impairments	1995	Steffenburg	Not interest of epilepsy in CP
17	Epilepsy in patients with spastic cerebral palsy: correlation with MRI findings at 5 years of age	1999	Akihisa Okumura	Study on a type of CP
18	Epilepsy in children with cerebral palsy	2003	GURURAJ	Duplicates further identified
19	Cause of death in cerebral palsy: a descriptive study	1999	Maudsley	Not interest of epilepsy in CP
20	Cerebral palsy	1994	Kuban	Not interest of epilepsy in CP
21	Trends in the prevalence of developmental disabilities in US children, 1997-2008	2011	Boyle	Not interest of epilepsy in CP
22	Trends in the prevalence of cerebral palsy in a population-based study	2002	Winter	Not interest of epilepsy in CP

23	Prevalence of cerebral palsy: Autism and Developmental Disabilities Monitoring Network, three sites, United States, 2004	2009	Arneson	Not interest of epilepsy in CP
24	The descriptive epidemiology of cerebral palsy	2006	Paneth	Not interest of epilepsy in CP
25	Decreasing prevalence in cerebral palsy: a multi-site European population-based study, 1980 to 2003	2016	SELLIER	no data
26	Birth Prevalence of Cerebral Palsy: A Population-Based Study	2016	Naarden Braun	Not interest of epilepsy in CP
27	An update on the prevalence of cerebral palsy: a systematic review and meta-analysis	2013	OSKOUI	Review
28	The descriptive epidemiology of cerebral palsy	2006	Panet	Not interest of epilepsy in CP
29	Cerebral palsy trends in Australia (1995-2009): a population-based observational study	2019	GALEA	no data
30	Prevalence of childhood disability in rural KwaZulu-Natal	2002	Couper	Not interest of epilepsy in CP
31	Epilepsy in patients with cerebral palsy	1997	Hadjipanayis	Duplicates further identified
32	Epidemiology of cerebral palsy	2006	Blair	no data
33	Etiology of cerebral palsy	2004	Meberg	Not interest of epilepsy in CP
34	Can we prevent cerebral palsy?	2003	Karin	Not interest of epilepsy in CP
35	The epidemiology of cerebral palsy: incidence, impairments and risk factors	2006	ODDING	no data
36	Changing panorama of cerebral palsy in Sweden. VIII. Prevalence and origin in the birth year period 1991-94	2001	Hagberg	No full text
37	SCPE work, standardization and definition--an overview of the activities of SCPE: a collaboration of European CP registers	2006	McManus	No full text
38	Prevalence of cerebral palsy in north-east Italy from 1965 to 1989	1999	Bottos	Not interest of epilepsy in CP
39	Early, Accurate Diagnosis and Early Intervention in Cerebral Palsy: Advances in Diagnosis and Treatment	2017	Novak,	Not interest of epilepsy in CP
40	Prevalence of cerebral palsy: Autism and Developmental Disabilities Monitoring Network, three sites, United States, 2004	2009	Carrie	Not interest of epilepsy in CP
41	Cerebral palsy	2016	Graham	Not interest of epilepsy in CP
42	Surveillance of cerebral palsy in Europe: a collaboration of cerebral palsy surveys and registers. Surveillance of Cerebral Palsy in Europe (SCPE)	2000		Not interest of epilepsy in CP
43	Cerebral palsy in Al-Quseir City, Egypt: prevalence, subtypes, and risk factors	2014	El-Tallawy	Duplicates further identified

44	Cerebral palsy: comprehensive review and update	2006	Jan	Not interest of epilepsy in CP
45	Prevalence of cerebral palsy in Turkish children between the ages of 2 and 16 years	2006	Serdaroglu	Not interest of epilepsy in CP
46	A special supplement: findings from the Australian Cerebral Palsy Register, birth years 1993 to 2006	2016	SMITHERS-SHEEDY	Not interest of epilepsy in CP
47	Prevalence of cerebral palsy in Turkish children between the ages of 2 and 16 years	2006	Serdaroğlu	Not interest of epilepsy in CP
48	Risk factors and clinical profiles in Turkish children with cerebral palsy: analysis of 625 cases	2008	Erkin	Not interest of epilepsy in CP
49	A review of the incidence and prevalence, types and aetiology of childhood cerebral palsy in resource-poor settings	2010	MGLADSTONE	Review
50	Nutritional Status of Children with Cerebral Palsy-Findings from Prospective Hospital-Based Surveillance in Vietnam Indicate a Need for Action	2019	Karim	included
51	Korean Database of Cerebral Palsy：A Report on Characteristics of Cerebral Palsy in South Korea	2017	Yim	included
52	Data on cerebral palsy in Vietnam will inform clinical practice and policy in low and middle-income countries	2021	Karim	Duplicates further identified
53	Nutritional status of children with cerebral palsy in remote Sumba Island of Indonesia: a community-based key informants study	2019	Jahan	Duplicates further identified
54	The Co-Occurrence of Autism Spectrum Disorder and Cerebral Palsy and Associated Comorbid Conditions in Children and Adolescents	2021	Leader	Not interest of epilepsy in CP
55	Epidemiology of Malnutrition among Children with Cerebral Palsy in Low- and Middle-Income Countries: Findings from the Global LMIC CP Register	2021	Jahan	Not interest of epilepsy in CP
56	Prevalence and Initial Diagnosis of Cerebral Palsy in Preterm and T erm-Born Children in Taiwan: A Nationwide, Population-Based Cohort Study	2021	Wang	Not interest of epilepsy in CP
57	Association of perinatal factors of epilepsy in very low birth weight infants, using a nationwide database in Japan	2019	Matsushita	Study on a type of CP
58	PAEDIATRIC CEREBRAL PALSY PREVALENCE AND HIGH-RISK FACTORS IN HENAN PROVINCE, CENTRAL CHINA	2019	Yuan	Not interest of epilepsy in CP
59	Incidence of Cerebral Palsy in Korea and the Effect of Socioeconomic Status: A Population-Based Nationwide Study	2018	Kim	Not interest of epilepsy in CP

60	Risk of cerebral palsy in Chinese children: A N:M matched case control study	2016	Gao	Not interest of epilepsy in CP
61	Trends in the prevalence of cerebral palsy in children born between 1988 and 2007 in Okinawa, Japan	2015	Touyama	Not interest of epilepsy in CP
62	A cross-sectional survey of growth and nutritional status in children with cerebral palsy in West China	2015	Wang	Not interest of epilepsy in CP
63	Prognostic Predictors for Ambulation in Thai Children With Cerebral Palsy Aged 2 to 18 Years	2015	Keeratisiroj	included
64	Estimating the prevalence of cerebral palsy in Taiwan: A comparison of different case definitions	2015	Chang	Not interest of epilepsy in CP
65	A review of the incidence and prevalence, types and aetiology of childhood cerebral palsy in resource-poor settings	2010	GLADSTONE	Reviews
66	Participation restriction of children with cerebral palsy living in Thailand and influential factors: A cross-sectional study	2022	Ploypetch	Not interest of epilepsy in CP
67	Epidemiology of cerebral palsy in low- and middle-income countries: preliminary findings from an international multi-centre cerebral palsy register	2021	Jahan	Not interest of epilepsy in CP
68	Prevalence and Initial Diagnosis of Cerebral Palsy in Preterm and T erm-Born Children in T aiwan: A Nationwide, Population-Based Cohort Study	2021	Wang	Not interest of epilepsy in CP
69	Temporal trends in perinatal mortality and cerebral palsy: A regional population-based study in southern Japan	2016	Kodama	Not interest of epilepsy in CP
70	Incidence patterns of cerebral palsy in Shiga Prefecture, Japan, 1977–1991	2002	Suzuki	Not interest of epilepsy in CP
71	Prevalence study of cerebral palsy in Hong Kong children	2006	Yam	Not interest of epilepsy in CP
72	Prevalence and lifetime healthcare cost of cerebral palsy in South Korea	2010	Park	Not interest of epilepsy in CP
73	Estimating the prevalence of cerebral palsy in Taiwan: A comparison of different case definitions	2014	Chang	Not interest of epilepsy in CP
74	Children with motor impairment related to cerebral palsy: Prevalence, severity and concurrent impairments in China	2016	He	Not interest of epilepsy in CP
75	Risk Factor of Mortality in Indonesian Children with Cerebral Palsy	2017	Gunawan	Not interest of epilepsy in CP
76	Post-discharge body weight and neurodevelopmental outcomes among very low birth weight infants in Taiwan: A nationwide cohort study	2018	Hsu	Not interest of epilepsy in CP
77	Incidence of Cerebral Palsy in Korea and the Effect of Socioeconomic Status: A Population-Based Nationwide Study	2018	Kim	Not interest of epilepsy in CP

78	Prolonged length of stay for acute hospital admissions as the increasing of age: A nationwide population study for Taiwan's patients with cerebral palsy	2018	Chiang	Not interest of epilepsy in CP
79	Cerebral palsy and developmental intellectual disability in children younger than 5 years: Findings from the GBD-WHO Rehabilitation Database 2019	2022	Olusanya	Not interest of epilepsy in CP
80	Serial Long-Term Growth and Neurodevelopment of Very-Low-Birth-Weight Infants: 2022 Update on the Korean Neonatal Network	2022	Jeon	Not interest of epilepsy in CP
81	Impact of Outborn/Inborn Birth Status of Infants Born at <29 Weeks of Gestation on Neurodevelopmental Impairment: A Nationwide Cohort Study in Korea	2022	Cho	Not interest of epilepsy in CP
